# Metabolic Reprogramming and Neurotransmitter Signaling Co-Option in the Glioma Immune Microenvironment: Dual-Axis Regulation of Immunosuppression

**DOI:** 10.3390/biom16070956

**Published:** 2026-06-28

**Authors:** Pengyu Zhao, Kamil Saramowicz, Angelika Adamus-Grabicka, Joanna Sikora, Wioletta Rozpędek-Kamińska

**Affiliations:** 1 Key Laboratory of Molecular Biophysics of the Ministry of Education, College of Life Science and Technology, Huazhong University of Science and Technology, Wuhan 430074, China; pengyu122601@hust.edu.cn; 2 Department of Clinical Chemistry and Biochemistry, Medical University of Lodz, 92-215 Lodz, Poland; kamil.saramowicz@stud.umed.lodz.pl; 3 Department of Bioinorganic Chemistry, Medical University of Lodz, 90-151 Lodz, Poland; angelika.adamus@umed.lodz.pl

**Keywords:** glioma, glioblastoma, tumor–immune microenvironment, metabolic reprogramming, immunometabolism, neurotransmitter signaling, microglia, lactate, immunosuppression

## Abstract

Glioma, particularly glioblastoma (GBM), is characterized by a strongly immunosuppressive tumor microenvironment that limits durable therapeutic responses. This review examines two interacting regulatory aspects of this microenvironment: metabolic reprogramming and neurotransmitter signaling co-option. Metabolic reprogramming is characterized by Warburg-type aerobic glycolysis, lactate accumulation, nutrient competition, and epigenetic lactylation, which generate an acidic and metabolically restrictive niche that impairs cytotoxic immune populations. In parallel, neurotransmitter signaling co-option, particularly through glutamatergic and GABAergic pathways, can influence neuron–glioma communication, microglial/macrophage phenotypes, and selected lymphocyte functions. As direct evidence for bidirectional interactions between metabolic reprogramming and neurotransmitter signaling in glioma remains incomplete, this relationship is presented as a working neurometabolic framework rather than a fully resolved mechanism. Lactate-driven immunometabolic suppression and glutamatergic neuron–glioma signaling currently have the strongest support from glioma-specific studies, whereas some GABAergic, serotonergic, and macrophage-metabolic mechanisms remain emerging or context-dependent. The review also considers how mechanism-guided patient stratification, metabolically optimized immunotherapy and mechanism-based combination strategies targeting defined metabolic and neurotransmitter pathways may help restore antitumor immune competence within the glioma microenvironment.

## 1. Introduction

Glioblastoma (GBM), the most common and aggressive primary malignant brain tumor in adults, is characterized by a dismal prognosis driven by relentless proliferation, diffuse infiltration, limited surgical resectability, and profound resistance to radiotherapy and chemotherapy. The limited efficacy of tumor-centric approaches has shifted attention toward the tumor microenvironment (TME), which is a central determinant of disease progression and therapeutic responsiveness [1]. The GBM TME is a heterogeneous landscape composed of malignant cells, resident and infiltrating immune cells, glioma-associated microglia/macrophages (GAMs), myeloid-derived suppressor cells (MDSCs), regulatory T cells (Tregs), exhausted T-cell subsets, astrocytes, neurons, extracellular matrix elements, and vascular barriers. A defining feature of this ecosystem is its profoundly immunosuppressive state; this is actively maintained by tumor-driven programs rather than by tumor expansion.

This review focuses on two key regulatory mechanisms. The first, metabolic reprogramming, supports tumor growth while imposing nutrient depletion, acidosis, and metabolite-driven suppression on immune cells. The second, based on endogenous neurotransmitter signaling and neuron–glia communication, can be recruited by gliomas arising within neural tissue to promote malignant behavior and reshape immune function. In the present review, these mechanisms are discussed as complementary layers of glioma immunobiology rather than as fully interchangeable processes. However, direct evidence for their bidirectional interaction in glioma remains incomplete [2,3,4,5].

In the present review, direct glioma or GBM data are distinguished from mechanistic insights derived from other tumor types or neuroinflammatory models. Accordingly, the term GBM is used for evidence derived specifically from glioblastoma studies, whereas glioma refers to mechanisms reported across diffuse gliomas or broader glioma datasets. The review first defines the immune landscape, then separately examines metabolic and neurotransmitter-associated mechanisms, and finally considers where the two may intersect.

The paper proposes a neurometabolic working framework that incorporates metabolic reprogramming and neurotransmitter signaling as interacting contributors to immune suppression within the glioma microenvironment. This framework is intended to support mechanistic interpretation and the development of therapeutic hypotheses, while recognizing that direct evidence for bidirectional interactions between these pathways remains incomplete.

## 2. Composition and Immunosuppressive Characteristics of the Glioblastoma Immune Microenvironment

### 2.1. Major Cellular Components and Their Functions in the Glioma Microenvironment

The glioma microenvironment represents a highly complex ecosystem composed of diverse cellular and non-cellular elements that collectively orchestrate tumor progression, immune evasion, and therapeutic resistance. At its core are malignant glioma cells, which function not merely as passive occupants but as active architects of their surrounding niche. These cells exhibit pronounced transcriptional heterogeneity and phenotypic plasticity, with distinct cellular states—including cycling, oligodendrocyte progenitor cell-like (OPC-like), neural progenitor cell-like (NPC-like), and mesenchymal-like (MES-like) subpopulations—identified through single-cell analyses [6]. This intrinsic heterogeneity is a major determinant of tumor molecular identity and critically shapes the composition and functional behavior of the surrounding microenvironment [7]. Glioma cells, particularly glioma stem cells, display potent tumorigenic and invasive capacities that support tumor growth and recurrence [8]. Glioma cells remodel the microenvironment through the secretion of signaling factors, metabolites, and extracellular vesicles (EVs). For example, GSC-derived EVs enriched with clusterin promote macrophage polarization toward an M2-like phenotype and contribute to temozolomide resistance [9]. Similarly, tumor-derived factors such as TGF-β1 and SPP1 actively recruit and reprogram stromal and immune populations within the glioma niche [6,10].

The most abundant non-neoplastic cellular component of the glioma microenvironment consists of tumor-associated macrophages and microglia (TAMs). In gliomas, TAM populations are predominantly skewed toward protumorigenic and immunosuppressive M2-like functional states, although their phenotypic spectrum varies across molecular glioma subtypes [11]. These cells promote tumor progression by secreting immunosuppressive cytokines, such as IL-10 and TGF-β; stimulating angiogenesis; and suppressing cytotoxic T-cell activity [12].

Recent observations have identified the presence of a distinct lipid-loaded TAM subset, referred to as tumor-associated foam cells (TAFs or TAFCs). They constitute a metabolically specialized protumorigenic population associated with unfavorable clinical outcomes and enhanced immunosuppressive remodeling of the TME [13,14]. Their differentiation is promoted by the scavenging of lipids carried by glioma-derived EVs [13]. Notably, the responsiveness of TAMs to therapeutic strategies such as CSF1R inhibition is strongly influenced by the genetic background of glioma, underscoring the context-dependent nature of macrophage–tumor interactions [11].

Tumor-infiltrating lymphocytes (TILs), including T cells and natural killer (NK) cells, are detectable within the glioma microenvironment but exhibit profound functional impairment. T cells frequently display an exhausted phenotype characterized by elevated expression of inhibitory receptors such as PD-1, reduced proliferative capacity, and diminished effector function, which is maintained by the surrounding suppressive cytokine milieu [12,15]. Importantly, even virus-specific memory T cells residing within glioma tissue may become functionally compromised [16].

The immunosuppressive landscape is supplemented by additional myeloid and lymphoid populations. Myeloid-derived suppressor cells (MDSCs) and regulatory T cells (Tregs) act as central regulators of immune inhibition within the glioma niche [17]. Recent evidence has also identified a highly suppressive neutrophil subset, disease-specific suppressive granulocytes (DSSGs), originating from skull bone marrow and meninges and correlating with glioma grade and adverse prognosis [18]. The metabolic or neurotransmitter-dependent regulation of DSSGs in glioma remains poorly characterized, and whether these cells participate in neurometabolic mechanisms of immune suppression remains to be determined.

Other stromal components further contribute to microenvironmental remodeling. Glioma-associated stromal cells (GASCs)—mesenchymal stromal-like populations functionally analogous to cancer-associated fibroblasts—localize predominantly within perivascular niches and promote angiogenesis, invasion, and tumor expansion [19,20]. Neurons are increasingly recognized as active participants in glioma progression, where tumor-induced alterations in neuronal activity contribute to pathological circuit remodeling and seizure susceptibility while simultaneously supporting tumor growth dynamics [21]. In parallel, non-cellular components, including extracellular matrix (ECM) remodeling and complex cytokine-chemokine signaling networks, establish a structural and biochemical scaffold that regulates cellular interactions, enhances invasion, and contributes to immune exclusion [22,23]. Collectively, this co-evolutionary interaction between heterogeneous glioma cells and diverse stromal and immune populations generates a profoundly immunosuppressive and tumor-supportive microenvironment that represents a major obstacle to effective therapeutic intervention [24,25].

### 2.2. Classical and Non-Classical Mechanisms of Glioma Immunosuppression

The immunosuppressive tumor microenvironment (TME) in gliomas is established through a coordinated interplay of classical and emerging non-classical regulatory mechanisms. Classical pathways include the upregulation of immune checkpoint ligands, the secretion of inhibitory cytokines, and the recruitment of regulatory immune populations that collectively suppress antitumor immunity. Glioblastoma cells express a broad spectrum of inhibitory checkpoint molecules, including PD-L1, PD-L2, and CTLA-4, which directly impair effector T-cell and natural killer (NK) cell activity [26]. Expression of these ligands is frequently enhanced in glioma stem-like cell populations, further reinforcing immune escape mechanisms [26]. In parallel, glioma-derived immunosuppressive cytokines such as TGF-β and IL-10 inhibit antigen presentation, promote regulatory T-cell differentiation, and drive polarization of myeloid populations—including TAMs and MDSCs—toward anti-inflammatory, tumor-supportive phenotypes [27]. Additionally, the structural and functional barrier imposed by the blood–brain barrier (BBB), together with the infiltrative growth pattern of glioblastoma, restricts access of both therapeutic agents and immune effector cells to tumor tissue, further limiting treatment efficacy [28]. Collectively, these classical mechanisms form a robust protective shield that contributes substantially to the limited clinical success of immune checkpoint blockade in glioblastoma patients [29].

More recently, non-classical mechanisms have emerged as critical regulators of glioma-associated immune suppression, reflecting the unique neurobiological context of tumors arising within the central nervous system. A central component of this regulatory layer involves active remodeling of neuronal circuitry by glioma cells, which generates regionally specialized immunosuppressive niches. Glioblastoma regions characterized by increased neuronal connectivity and synaptogenic activity display an enrichment of anti-inflammatory TAM populations and depletion of pro-inflammatory immune subsets [30,31]. This process is mediated in part by synaptogenic factors such as thrombospondin-1 (TSP-1); genetic deletion of TSP-1 suppresses neuron–glioma synaptic signaling while restoring antigen presentation capacity and pro-inflammatory immune activation, resulting in prolonged survival in immunocompetent but not immunodeficient mouse models [30,31]. Pharmacological inhibition of glutamatergic neuronal hyperexcitability similarly redirects TAM populations toward less immunosuppressive functional states, highlighting a direct mechanistic connection between neuronal activity and immune regulation [30].

Beyond neuronal interactions, bidirectional communication with other CNS-resident cells—particularly astrocytes—represents an additional layer of immune modulation. Glioblastoma cells can induce astrocytes to adopt immunosuppressive phenotypes through IL-11/STAT3-dependent TRAIL expression, leading to apoptosis of infiltrating T cells [32]. Similarly, ANXA1-FPR1 signaling between astrocytes and glioma cells suppresses immunogenic tumor cell death and dampens inflammatory signaling within astrocytes, thereby establishing a protective tumor-supportive niche [33]. Recent spatial analyses in oral squamous cell carcinoma further show that ANXA1-FPR signaling can organize spatially restricted MDSC-mediated immune suppression, supporting the broader relevance of ANXA1-FPR programs as cellular regulators of suppressive myeloid niches, although this specific mechanism still requires validation in glioma [34]. Tumor-derived extracellular vesicles (EVs) represent another potent class of non-classical mediators capable of disseminating immunosuppressive signals both locally and systemically. EVs released from IDH-mutant gliomas carry molecular cargo that reduces tumor-infiltrating lymphocyte and dendritic cell abundance while increasing circulating monocyte populations, thereby accelerating tumor progression [35]. Glioma-derived EVs additionally impair dendritic cell antigen presentation, promote polarization of TAMs and MDSCs, and reinforce T-cell exhaustion programs within the tumor microenvironment [28,36].

**Figure 1:**
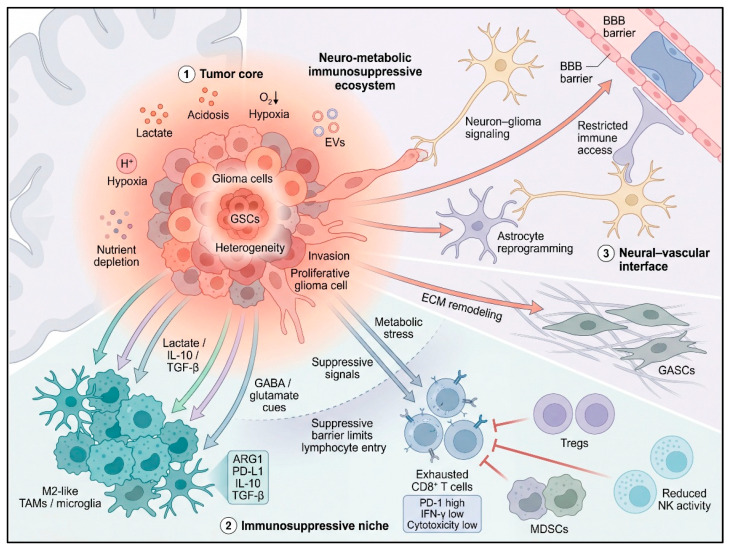
The glioma immune microenvironment as a neurometabolic immunosuppressive ecosystem. Heterogeneous glioma cells and GSCs generate a metabolically stressed niche marked by lactate accumulation, acidosis, hypoxia, EV release and nutrient depletion. These cues cooperate with neuron–glioma signaling, astrocyte reprogramming, ECM remodeling and BBB restriction to promote suppressive TAMs/microglia, exhausted CD8^+^ T cells, Tregs, MDSCs and impaired NK cells. Created in BioRender. Rozpędek-Kamińska, W. (2026) https://BioRender.com/wjiutpa, accessed on 28 June 2026.

Metabolic rewiring of tumor and immune compartments represents another central non-classical axis of immune suppression. The oncometabolite R-2-hydroxyglutarate (R-2HG), produced in IDH-mutant gliomas, reprograms tryptophan metabolism in infiltrating myeloid cells through activation of the aryl hydrocarbon receptor, thereby establishing dynamic immunosuppressive myeloid states that limit effective T-cell responses [37]. R-2HG additionally suppresses inflammatory activation of microglia through inhibition of the FTO/NF-κB signaling pathway [38]. Tumor-derived lactate further contributes to immune suppression by reducing infiltration and functional activity of CD8^+^ T cells within the glioma microenvironment [39]. Together, these non-classical regulatory pathways—including neuron–glioma–immune crosstalk, astrocyte reprogramming, vesicle-mediated communication, and metabolic remodeling—constitute a coordinated network of mechanisms that are increasingly recognized as central drivers of glioma-associated immune evasion and therapeutic resistance. These cellular, stromal, metabolic and neurobiological components define the glioma immune microenvironment as a spatially organized neurometabolic immunosuppressive ecosystem (Figure 1).

## 3. Metabolic Reprogramming of Glioma Cells: Shaping an Immunosuppressive Metabolic Microenvironment

### 3.1. Warburg Effect and Lactate-Mediated Acidosis

Glioma cells, like many malignant cell types, undergo profound metabolic reprogramming characterized by preferential reliance on glycolysis for energy production even in the presence of sufficient oxygen, a phenomenon known as the Warburg effect or aerobic glycolysis [40]. This metabolic adaptation is driven by oncogenic signaling networks and results in inefficient ATP generation accompanied by sustained lactate production, in contrast to the oxidative phosphorylation predominantly utilized by surrounding neuronal tissue [41]. Importantly, glycolytic rewiring in glioma cells supports not only bioenergetic requirements but also anabolic demands by supplying metabolic intermediates necessary for nucleotide, lipid, and amino acid biosynthesis required for rapid proliferation [42]. This metabolic phenotype represents a defining feature of gliomas, including glioblastoma (GBM), and can be directly visualized using advanced metabolic imaging approaches. For example, dynamic oxygen-17 MRI has demonstrated a reduced cerebral metabolic rate of oxygen (CMRO_2_) within glioma regions relative to normal brain tissue, providing in vivo evidence of glycolytic metabolic dominance [43]. Similarly, hyperpolarized 13C MRI using tracers such as [1-^13^C]pyruvate enables real-time detection of enhanced pyruvate-to-lactate conversion in high-grade gliomas, correlating with tumor aggressiveness and spatial metabolic heterogeneity [44]. At the molecular level, this metabolic shift is supported by regulators including pyruvate kinase M2 (PKM2), whose expression promotes glycolytic flux and represents a potential therapeutic target [45]. In addition, Galectin-1 (Gal-1) reinforces the Warburg phenotype in glioma stem cells, and its depletion partially restores oxidative metabolism while reducing lactate accumulation [46]. Collectively, these observations identify aerobic glycolysis as a central metabolic adaptation sustaining glioma growth and shaping the surrounding immune microenvironment.

Enhanced glycolytic flux results in excessive extracellular lactate accumulation mediated by monocarboxylate transporters such as MCT1 and MCT4 [47]. This lactate efflux leads to progressive acidification of the tumor microenvironment (TME), generating a condition commonly referred to as lactic acidosis [3]. Acidification of the glioma niche functions as a potent immunosuppressive barrier by directly impairing cytotoxic immune cell activity. Reduced extracellular pH inhibits proliferation, migratory capacity, and effector function of cytotoxic T lymphocytes and natural killer (NK) cells, thereby attenuating antitumor immune responses [48,49]. Acidic conditions further disrupt immune synapse formation between tumor and immune cells, driving tumor-infiltrating lymphocytes toward functionally inactive or anergic states [50]. In parallel, extracellular acidosis promotes the differentiation and suppressive activity of regulatory T cells (Tregs), reinforcing immune tolerance within the glioma microenvironment [51]. Notably, transient exposure to acidic conditions can induce durable metabolic reprogramming in Tregs, sustaining their suppressive phenotype even after removal from the acidic niche [52]. These findings indicate that lactate-driven acidification represents an active regulatory mechanism of immune evasion rather than a passive metabolic by-product. Consistently, studies in lung cancer models demonstrate that enzymes promoting lactate export and extracellular acidification, including TMPRSS11B, generate macrophage-enriched immunosuppressive microenvironments dominated by M2-like populations, further supporting the mechanistic link between lactate-dependent acidosis and immune suppression [53]. Thus, extracellular acidification constitutes a core metabolic strategy through which gliomas neutralize antitumor immune surveillance.

Beyond its role in extracellular acidification, lactate functions as a signaling metabolite capable of directly reprogramming immune cell phenotypes within the tumor microenvironment. Lactate signaling occurs in part through receptors such as the G-protein-coupled receptor GPR81 [54], contributing to polarization of tumor-associated macrophages toward anti-inflammatory and tumor-supportive M2-like states [55]. These macrophage populations promote angiogenesis, extracellular matrix remodeling, and the suppression of adaptive immune responses. Lactate-dependent environmental acidification additionally alters macrophage circadian regulation, further contributing to the functional dysregulation of tumor-associated macrophages within glioma tissue [56]. Importantly, lactate also mediates epigenetic regulation through histone lactylation, a post-translational modification linking metabolic flux directly to transcriptional control [40]. In gliomas, histone lactylation at residues including H3K18 and H4K8 promotes expression of oncogenic programs associated with proliferation, radioresistance, and cytoprotective autophagy [57,58]. For example, lactylation of histone H4 at lysine 8 (H4K8la) activates transcription of the autophagy regulator NUPR1, enhancing glioma cell survival under stress conditions [58]. This lactate-driven metabolic–epigenetic regulatory axis integrates extracellular acidification with transcriptional reprogramming of both tumor and immune compartments toward immunosuppressive states [3]. Accordingly, therapeutic strategies targeting lactate production, transport, receptor signaling, or downstream lactylation pathways have emerged as promising approaches for restoring immune competence within the glioma microenvironment and improving responses to immunotherapy [59,60].

### 3.2. Nutrient Competition and Immune Cell Functional Exhaustion

The immunosuppressive tumor microenvironment (TME) in glioma is strongly shaped by intense metabolic competition between tumor cells and infiltrating immune populations for essential nutrients required to sustain cellular activation and effector function. A central component of this competition involves the preferential uptake of glucose by glioma cells, which maintain elevated glycolytic flux even under normoxic conditions. This metabolic dominance generates a state of severe glucose deprivation within the tumor niche [61]. Effector T cells depend critically on glycolytic metabolism to support activation, clonal expansion, cytokine production (including IFN-γ secretion), and cytotoxic granule release containing perforin and granzymes [62]. Consequently, glucose restriction within the glioma microenvironment induces metabolic stress in infiltrating T cells, leading to impaired proliferation, reduced cytokine production, and diminished cytotoxic capacity [63]. This resource competition, therefore, represents an active immune evasion strategy rather than a passive consequence of tumor growth, reflecting direct metabolic antagonism between proliferating tumor cells and antitumor immune populations [64].

Competition for amino acids constitutes an additional layer of metabolic immune suppression. A key regulatory pathway involves upregulation of indoleamine-2,3-dioxygenase 1 (IDO1), which catalyzes tryptophan catabolism [65]. However, tryptophan 2,3-dioxygenase 2 (TDO2) is also independently expressed in GBM and may constitute a mechanistically distinct source of kynurenine production and immunosuppressive signaling [66]. Depletion of extracellular tryptophan activates stress-response pathways such as GCN2 kinase signaling in T cells, thereby inhibiting proliferation and effector differentiation [65]. Simultaneously, the accumulation of downstream metabolites such as kynurenine (KYN) promotes apoptosis of effector T cells and enhances the expansion of regulatory T cells (Tregs), further reinforcing immune tolerance [61]. This coordinated mechanism—combining nutrient depletion with production of immunoregulatory metabolites—establishes a potent tryptophan-dependent immunosuppressive axis within the glioma microenvironment [67].

**Figure 2:**
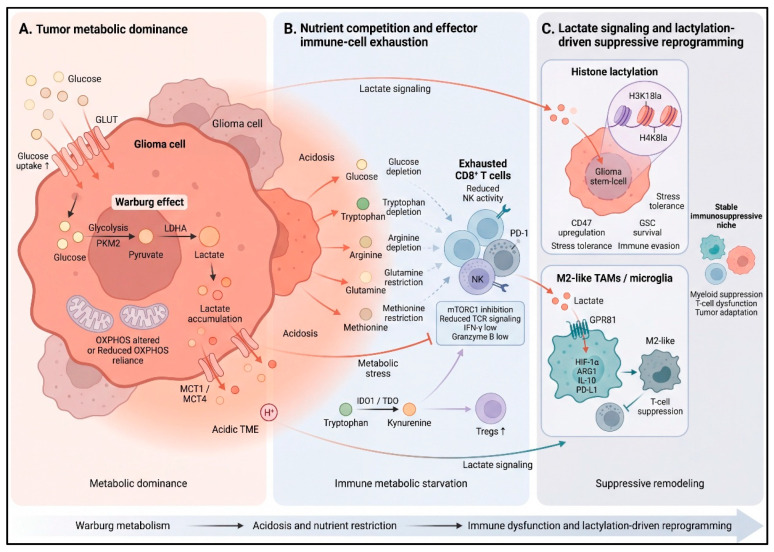
Metabolic reprogramming generates an acidic, nutrient-restricted and epigenetically suppressive glioma niche. Warburg-like glycolysis and MCT1/MCT4-mediated lactate export drive acidosis, while competition for glucose, tryptophan, arginine, glutamine and methionine limits cytotoxic immune function. Lactate signaling and histone lactylation further promote suppressive TAM/microglial programs and glioma immune evasion. Created in BioRender. Rozpędek-Kamińska, W. (2026) https://BioRender.com/wjiutpa, accessed on 28 June 2026.

Glioma-associated metabolic competition extends beyond tryptophan to additional amino acid pathways. Enhanced glutamine consumption by tumor cells restricts the metabolic flexibility of immune populations, including dendritic cells and macrophages, whose activation states depend on glutaminolysis-dependent signaling pathways [68]. Similarly, dysregulated arginine metabolism contributes to immune suppression through elevated expression of arginase-1 (ARG1) in both glioma cells and tumor-associated myeloid populations, resulting in depletion of L-arginine required for T-cell and natural killer (NK) cell proliferation and cytotoxic function [69]. Together, these coordinated metabolic constraints generate a nutrient-restricted microenvironment that drives functional exhaustion and anergy in effector immune populations. This metabolically hostile niche represents a major obstacle to immunotherapeutic efficacy and highlights the importance of strategies aimed at restoring the metabolic fitness of immune cells within the glioma TME [70,71]. The major mechanisms by which glioma metabolic reprogramming generates an acidic, nutrient-restricted and epigenetically suppressive immune niche are summarized in Figure 2.

## 4. Co-Option of Neurotransmitter Signaling Networks: Glioma Exploits the Brain’s Chemical Language

### 4.1. Abnormal Activation and Co-Option of the Glutamate Signaling Pathway

Glioma cells actively manipulate the brain’s excitatory neurotransmitter system through the overexpression of glutamate transport machinery, most notably the cystine-glutamate antiporter xCT (SLC7A11), which constitutes the catalytic subunit of system Xc^−^ [72]. This dysregulation drives excessive glutamate efflux into the extracellular tumor microenvironment (TME) [73]. The resulting accumulation of extracellular glutamate is a defining feature of the glioma niche and exerts multiple protumorigenic effects [74]. Importantly, glutamate release is not a passive metabolic consequence but a tightly regulated component of glioma biology. Loss of the transcriptional repressor Capicua (CIC), which is frequently mutated in oligodendrogliomas, increases xCT/SLC7A11 expression and further elevates extracellular glutamate levels [73]. Conversely, the tumor suppressor p53 functions as a negative transcriptional regulator of SLC7A11, and its loss or mutation, common in glioblastoma, promotes increased xCT expression and glutamate release [75]. Together, these alterations establish a dysregulated glutamatergic feed-forward circuit that supports glioma progression.

Within the TME, elevated glutamate acts in both autocrine and paracrine modes through the activation of ionotropic glutamate receptors, including AMPA and NMDA receptors, expressed on glioma cells themselves [76]. Activation of these receptors, particularly AMPA receptors (AMPARs), critically supports tumor proliferation, invasion, and survival [77]. This signaling frequently occurs through neuron-to-glioma synaptic structures, in which presynaptic neurons release glutamate onto postsynaptic-like domains on glioma cells, thereby forming functional “neurogliomal synapses” [76,78]. The resulting membrane depolarization and calcium influx activate downstream oncogenic signaling pathways involving Src kinase, PDGFRα, and the RAS/ERK cascade, which directly promote tumor growth [79]. This mode of synaptic communication effectively integrates glioma cells into existing neural circuitry, enabling them to exploit activity-dependent neuronal signaling for malignant advantage [80]. The biological significance of this pathway is underscored by observations linking high functional connectivity within gliomas to reduced patient survival [76].

Beyond its direct effects on tumor cells, excessive glutamate exerts marked excitotoxic effects on the surrounding neural tissue [72]. This excitotoxicity contributes to neurodegeneration, neuronal loss, and local tissue disruption, thereby creating physical and functional conditions favorable for tumor expansion [76]. Moreover, the resulting disturbance of the excitatory–inhibitory balance promotes a hyperexcitable microenvironment, which is strongly associated with the high prevalence of tumor-associated epilepsy [81,82]. Peritumoral hyperexcitability and seizure susceptibility arise from complex host–tumor interactions that include loss of inhibitory interneurons and reactive alterations in glial populations [83]. Glutamatergic signaling also modulates the broader tumor microenvironment by acting on non-neoplastic cellular compartments. Microglia and macrophages express metabotropic glutamate receptors (mGluRs), and glutamate stimulation can reshape their inflammatory phenotype [84]. In particular, excessive extracellular glutamate promotes polarization of tumor-associated macrophages toward anti-inflammatory, protumorigenic states, thereby reinforcing immune suppression [74]. Taken together, this multifaceted co-option of glutamatergic signaling—simultaneously driving tumor growth, remodeling neuronal function, and reprogramming immune responses—defines a central and therapeutically actionable axis of glioma biology [85,86].

### 4.2. Role of GABAergic Signaling in the Glioma Immune Microenvironment

Gamma-aminobutyric acid (GABA), the principal inhibitory neurotransmitter of the brain, exerts a multifaceted role within the glioma immune microenvironment that extends well beyond neuronal signaling to directly influence tumor behavior and immune escape. Glioma cells actively participate in shaping the local GABAergic milieu. They are capable of synthesizing and secreting GABA or modulating its availability through the uptake of precursor substrates, thereby influencing the concentration of this key signaling molecule within the tumor niche [87]. This active remodeling of the neurotransmitter environment represents an important component of the broader neural–immune–tumor axis. Multi-omics analyses of adult gliomas derived from The Cancer Genome Atlas (TCGA) have identified tumor subgroups defined by neurotransmitter-related gene expression signatures, underscoring the functional relevance of neurotransmitter signaling pathways, including GABAergic signaling, in glioma biology [88]. Aberrant expression of GABA system components is not merely an epiphenomenon but is closely linked to malignant progression. For example, the GABA-A receptor π subunit (GABRP) is frequently overexpressed in gliomas and other cancers, and elevated expression correlates with poorer patient prognosis, suggesting a direct contribution to tumor aggressiveness [89]. Functionally, GABA signaling promotes glioma cell proliferation. Studies using GABRP knockdown in U87 and U251 glioma cell lines demonstrated marked suppression of proliferation and migration, implicating GABA-A receptor-mediated signaling, potentially through PI3K/AKT-associated pathways, in maintenance of tumor growth [89]. This autocrine and paracrine loop enables glioma cells not only to respond to a GABA-enriched niche but also to actively sustain it.

The immunomodulatory consequences of GABAergic signaling are substantial and represent a major mechanism by which gliomas consolidate an immunosuppressive tumor microenvironment (TME). GABA exerts inhibitory effects on immune cell function primarily through GABA-A and GABA-B receptors expressed on multiple immune populations. In T cells, GABA signaling suppresses cytotoxic effector function, and this inhibition can be partially reversed by pharmacological blockade, as illustrated by amentoflavone, which partially counteracts GABA-mediated T-cell suppression through modulation of GABA-A receptor-associated signaling in preclinical models [89]. Beyond adaptive immunity, GABAergic signaling critically influences innate immune populations, particularly macrophages, promoting their polarization toward anti-inflammatory, M2-like tumor-supportive phenotypes. Bioinformatic analyses consistently demonstrate that elevated expression of GABA-related genes, including GABRP, is associated with increased infiltration of immunosuppressive cell populations within the TME, including regulatory T cells (Tregs) and M2 macrophages [89]. This coordinated recruitment and polarization establishes a robust barrier to effective antitumor immunity. Additional support for the link between neurotransmitter signaling and immune architecture derives from TCGA-based analyses demonstrating significant differences in inflammatory microenvironmental composition among neurotransmission-defined glioma subgroups, with selected categories exhibiting altered representation of M2 macrophage populations [88].

**Figure 3:**
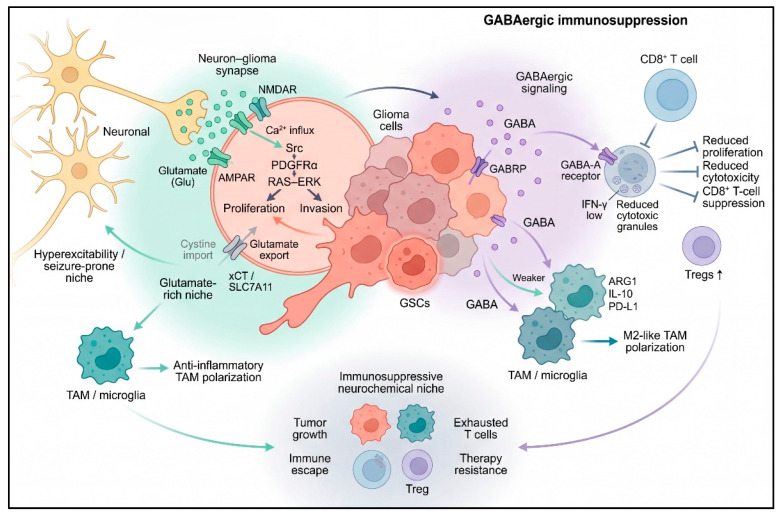
Glioma co-opts glutamatergic and GABAergic signaling to reshape tumor–neuron–immune communication. xCT/SLC7A11-mediated glutamate export and neuron–glioma synapses activate AMPAR/NMDAR-dependent pathways that support proliferation, invasion and neural hyperexcitability. GABAergic signaling suppresses CD8^+^ T-cell activity and promotes M2-like TAM polarization, linking neurochemical remodeling to immune escape. Created in BioRender. Rozpędek-Kamińska, W. (2026) https://BioRender.com/wjiutpa, accessed on 28 June 2026.

The cellular origins of these immunosuppressive signals are likely heterogeneous. For instance, translocator protein (TSPO) expression in glioblastoma identifies aggressive mesenchymal-like tumor populations associated with increased infiltration of tumor-associated macrophages, which serve as major executors of immunosuppressive programs potentially influenced by GABAergic cues [90]. Moreover, observations from neuroinflammatory disorders such as antibody-associated autoimmune encephalitis, in which GABA receptors are directly targeted, highlight the broader importance of astrocytes and microglia in neuroimmune communication [91]. Although glioma cells may represent the dominant source of GABA-related immunoregulatory signaling in the tumor setting, reactive astrogliosis and microglial activation are pervasive features of glioma pathology, and direct GABA-mediated effects on these glial compartments likely further reinforce the suppressive microenvironment. Thus, glioma cells exploit the endogenous GABAergic system to achieve a dual outcome: direct support of tumor proliferation and simultaneous suppression of antitumor immunity through inhibition of cytotoxic T cells and promotion of macrophage polarization toward M2-like states. This coordinated action positions GABAergic signaling as a central regulatory pillar of the immunosuppressive glioma microenvironment and a compelling therapeutic target for strategies aimed at restoring immune competence [87,89]. These glutamatergic and GABAergic mechanisms together illustrate how glioma cells co-opt the brain’s neurochemical language to coordinate tumor growth, neural remodeling and immune suppression (Figure 3).

## 5. Crosstalk Between Metabolites and Neurotransmitters: A Working Model and Evidence Gaps

### 5.1. Interaction Between Lactate and Neurotransmitter Systems

Direct mechanistic evidence that lactate and neurotransmitter pathways bidirectionally regulate one another in glioma remains limited. Current support is best viewed in three tiers. First, glioma tissues and experimental glioma models show measurable alterations in lactate, glutamate, glutamine, and GABA pools, indicating that tumor metabolism and neurotransmitter-associated pathways are spatially and biochemically connected [92,93,94,95]. Second, astrocytes provide a plausible mechanistic bridge because they regulate glutamate–glutamine cycling and are sensitive to metabolic stress, acidosis, and glioma-derived factors. Third, both lactate-rich metabolic niches and abnormal neurotransmitter signaling can affect immune cell function, particularly myeloid polarization and T-cell fitness. These observations support a testable model of neurometabolic coupling, but they do not yet prove a fully reciprocal regulatory circuit in human glioma.

Lactate may influence neurotransmitter availability through proton-coupled monocarboxylate transport, intracellular pH changes, and altered astrocytic metabolism. MRS-based studies in glioma patients and glioma-bearing animals provide evidence of altered lactate and neurotransmitter-related metabolites, while multi-omics analyses identify perturbations in alanine, aspartate, and glutamate metabolism [92,93,94,95]. These data are valuable for mapping metabolic–neurochemical association, but they are largely correlative. Therefore, statements regarding lactate-driven changes in glutamate or GABA homeostasis should be interpreted as mechanistic hypotheses requiring direct testing using cell-type-resolved and spatially controlled models [96].

Within this cautious framework, tumor-derived lactate, acidosis, and nutrient competition may disturb neurotransmitter balance, while altered glutamate and GABA signaling may secondarily influence tumor and immune cell metabolism. The strongest glioma-specific evidence currently links glutamate release and neuron–glioma synaptic activity to tumor growth, neural hyperexcitability, and immune remodeling. By contrast, the extent to which lactate directly regulates neurotransmitter synthesis or clearance in glioma-associated astrocytes, microglia, or immune infiltrates remains insufficiently defined. Thus, this section proposes an integrated model of metabolic–neurotransmitter interaction while explicitly recognizing that direct bidirectional co-regulation remains an emerging area rather than an established conclusion [92,93,96].

### 5.2. Regulation of Immune Cell Metabolism by Neurotransmitters

Neurotransmitter signaling can influence immune cell metabolism, but the strength of evidence varies by neurotransmitter, cell type, and tumor context. GABA provides an instructive example. Experimental work outside glioma shows that GABA can reshape macrophage metabolism, inflammasome activity, and polarization through pathways involving succinate-FAD-LSD1 signaling, OXPHOS, and methylation-linked metabolic regulation [97,98]. In cancer models, tumor-derived GABA or GABA-related metabolites can suppress CD8^+^ T-cell activation and support immune evasion [99,100,101]. These studies establish biological plausibility, but they should not be interpreted as direct proof that identical GABA-macrophage mechanisms operate in glioma.

Glutamate and serotonin illustrate a similar principle. Glutamate availability is closely linked to glutamine metabolism and receptor-mediated calcium signaling, and glioma datasets suggest associations between amino acid transporters and immune infiltration [102]. Serotonin-dependent GAPDH serotonylation in CD8^+^ T cells promotes a glycolytic shift that supports antitumor immunity [103], and serotonergic signaling can modulate macrophages, dendritic cells, and PD-L1 expression in other tumor contexts [104]. However, the relevance of GAPDH serotonylation to the glioma TME remains unproven. We therefore cite this mechanism as an example of how neurotransmitter-derived metabolites can regulate immune metabolism, not as a glioma-validated pathway [99].

**Figure 4:**
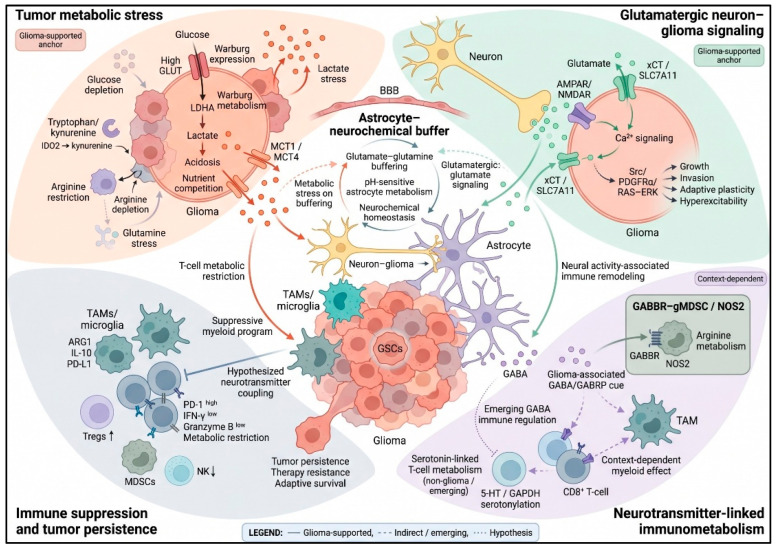
Metabolite–neurotransmitter crosstalk shapes glioma-associated immunosuppression. Glioma metabolic stress, including lactate accumulation, acidosis and nutrient competition, cooperates with neurotransmitter signaling co-option to reshape the immune microenvironment. Glutamatergic neuron–glioma signaling and xCT/SLC7A11-mediated glutamate export support tumor growth and neural hyperexcitability, whereas GABA-associated and GABBR–gMDSC/NOS2 pathways may contribute to immune metabolic suppression in a context-dependent manner. Astrocytes may serve as an intermediate buffering interface linking metabolic stress to neurochemical imbalance. Solid, dashed and dotted arrows indicate glioma-supported, emerging and hypothesis-generating mechanisms, respectively. Created in BioRender. Rozpędek-Kamińska, W. (2026) https://BioRender.com/wjiutpa, accessed on 28 June 2026.

The most direct glioma-relevant anchor for neurotransmitter-mediated immunometabolism is the sex-specific role of GABA receptor B (GABBR) signaling in female granulocytic MDSCs, where enhanced L-arginine metabolism and nitric oxide synthase 2 (NOS2) expression promote GBM growth in an immune-dependent manner [105]. This observation connects neurotransmitter signaling, immune cell metabolism, and glioma progression more directly than many adjacent models and suggests that sex and myeloid subtype may be important variables in future therapeutic stratification. Systems-level analyses of chronic psychological stress and oncogenesis also support the broader concept that neural and neuroendocrine states can modulate tumor immunity and metabolism, although these data are not glioma-specific [106]. Overall, neurotransmitter-dependent regulation is best interpreted as an emerging layer of the glioma immune microenvironment, with strong rationale but uneven mechanistic validation across pathways (Figure 4) [107,108,109].

## 6. Microglia and Macrophages at the Metabolic–Neural Interface

### 6.1. Metabolic Constraints on TAM/Microglial Phenotypes

The metabolic state of tumor-associated macrophages/microglia (TAMs) within the glioma microenvironment is tightly linked to their functional phenotype. Because lactate-driven acidosis and nutrient competition were discussed above, this section focuses on how myeloid cells adapt metabolically once they enter the glioma niche. TAMs integrate hypoxia, glucose restriction, amino acid availability, and lipid-rich signals through coordinated changes in glycolysis, oxidative phosphorylation (OXPHOS), fatty acid oxidation (FAO), the tricarboxylic acid cycle, and autophagy [110,111,112]. Comparable neuroinflammatory settings, such as post-stroke inflammation, also show that microglia/macrophage polarization is coupled to temporal changes in glycolysis, lactate handling, mitochondrial metabolism, and inflammatory resolution, providing a useful energy-metabolism framework while remaining disease-context distinct from glioma [113,114,115].

In glioma, these metabolic adaptations help TAMs survive in hypoxic and nutrient-poor regions and maintain angiogenic, immunosuppressive, and therapy-resistant functions [110,112]. Tumor-derived metabolites such as lactate and kynurenine can act as instructive signals, but their effects should be interpreted in a spatial and cell-state-specific manner rather than as a uniform M2 switch. For example, tryptophan catabolism may simultaneously affect tumor cells, myeloid cells, and T cells through kynurenine–AhR signaling, whereas lipid uptake can generate metabolically specialized foam-like TAM states. Thus, TAM metabolism represents a convergence point through which the tumor niche translates metabolic stress into suppressive myeloid function [115,116,117].

The key unresolved issue for the dual-axis model is how these metabolic programs are modified by neural activity and neurotransmitter exposure in glioma. Current evidence indicates that myeloid phenotypes vary across spatial niches and can be influenced by neuron–glioma signaling, but direct mapping of neurotransmitter cues onto TAM mitochondrial state, FAO, or amino acid metabolism remains incomplete. Future studies combining spatial metabolomics, single-cell myeloid profiling, and neural-activity mapping will be necessary to determine whether neural cues actively instruct TAM metabolism or mainly act in parallel with metabolic stress.

### 6.2. Neurotransmitter Signaling and Myeloid Immune Regulation

Microglia and macrophages express neurotransmitter receptors and can respond to neural cues within the glioma microenvironment. Evidence most directly relevant to glioma indicates that neuron–glioma signaling and glutamatergic activity can shape regional immune states, including TAM phenotypes and antigen presentation capacity [30,31]. These observations support a neural contribution to myeloid regulation, but the molecular pathways linking neurotransmitter exposure to specific metabolic programs in glioma-associated TAMs remain only partly defined [88,118].

GABAergic mechanisms are particularly important to discuss with caution. Tumor-derived GABA has been shown in non-glioma tumor models to act on macrophages and promote M2-associated markers such as Arg1 and IL-10 [118]. GAD1 expression and GABA-related pathways are also observed in glioma datasets [88], and GABBR signaling in female gMDSCs provides a glioma-relevant example of neurotransmitter-linked myeloid metabolism [105]. Nevertheless, the conclusion that GABA directly drives macrophage polarization in glioma remains inferential and requires direct validation in glioma microglia/macrophage systems [104,119,120,121].

Other neurotransmitter systems may also influence myeloid cells. Glutamate signaling through metabotropic receptors can suppress inflammatory activation in microglia, whereas NMDAR activation on tumor-associated macrophages may enhance calcium influx, reactive oxygen species production, and immunosuppressive activity [119,122]. Dopaminergic, serotonergic, and glycinergic pathways provide additional examples of neurotransmitter-dependent myeloid regulation, but most supporting data come from non-glioma or broader inflammatory settings [104,120,121]. Accordingly, neurotransmitter receptor targeting should be presented as a promising but still early-stage strategy for myeloid reprogramming in glioma rather than as a clinically established approach.

## 7. Dual Inhibitory Mechanisms on Effector T-Cell Function

### 7.1. Metabolic Deprivation and Dysfunction

Effector T cells within the glioma microenvironment are exposed to intense competition for critical nutrients, including glucose and selected amino acids, resulting in a profound inability to sustain effective glycolysis and oxidative metabolism. This bioenergetic crisis culminates in functional impairment and, in some settings, apoptotic loss. The highly glycolytic phenotype of glioma cells, driven in part by factors such as hypoxia-inducible factor 1α (HIF-1α), generates a nutrient-depleted and acidic tumor microenvironment (TME) [123]. This metabolic niche is further characterized by the accumulation of by-products such as lactic acid, which amplifies local immune suppression [124]. Glioma cells and glioma stem cells (GSCs) also display marked metabolic flexibility, allowing them to persist under glucose-restricted conditions by using alternative carbon sources such as lactate and acetate, thereby maintaining their aggressive phenotype [125]. This metabolic adaptability supports tumor survival while simultaneously intensifying the hostile conditions encountered by infiltrating immune cells. More broadly, the glioma TME is recognized as a metabolically disadvantaged compartment in which impaired vascular exchange and sustained tumor cell metabolism drive hypoxia, nutrient depletion, and waste product accumulation [126]. This hostile biochemical landscape imposes substantial constraints on tumor-infiltrating lymphocytes (TILs), including CD8^+^ T cells. Direct competition for metabolic substrates such as glucose renders T cells dysfunctional or exhausted, as tumor cells generally outcompete them for these shared resources [63]. Nutrient depletion, particularly involving glucose as well as amino acids such as arginine and methionine, therefore represents a central mechanism by which the glioma TME suppresses antitumor immunity [127]. For example, glioma cells exhibit dominant methionine metabolism, which is associated with a more immunosuppressive microenvironment, including an increased infiltration of M2-type macrophages [128]. Likewise, arginine metabolic reprogramming in glioma cells reduces arginine availability in the TME and thereby suppresses T-cell proliferation, activation, and effector function [129]. Importantly, this metabolic competition extends beyond passive starvation, as tumor-derived factors and metabolic enzymes also reshape the surrounding chemokine and cytokine milieu, further weakening recruitment and functionality of cytotoxic T lymphocytes and natural killer cells while favoring accumulation of immunosuppressive populations [130].

The acidic environment generated by glycolytic tumor metabolism imposes an additional layer of T-cell dysfunction. Acidosis suppresses T-cell receptor (TCR) signaling and inhibits mTORC1 activity, a central regulator of T-cell activation, proliferation, and differentiation [131]. As a direct consequence of enhanced glycolysis and lactate secretion, the acidic TME also remodels the extracellular matrix and generates metabolic gradients that may support metabolic cooperation among tumor cell populations while remaining detrimental to immune cell fitness [123]. Lactic acid itself acts as a potent immunosuppressive metabolite. In addition to driving extracellular acidification, lactate directly inhibits cytolytic activity and cytokine production, including IFN-γ and TNF-α secretion, in CD8^+^ T cells [124]. Lactate accumulation may also support expansion and functional activity of regulatory T cells (Tregs), thereby reinforcing suppression of effector immunity [130]. This multilayered metabolic inhibition extends beyond T cells. Dendritic cell (DC) function is also impaired in the glioma TME, while glioma-derived exosomes with reduced MHC-I expression contribute to CD8^+^ T-cell dysfunction and systemic immune escape [132,133]. Persistent metabolic stress additionally drives epigenetic remodeling in T cells. Prolonged exposure to nutrient-poor and metabolically restrictive conditions can result in loss of histone methyltransferase EZH2 in tumor-infiltrating lymphocytes, leading to mitochondrial dysfunction and establishment of an exhausted phenotype [134]. In this setting, metabolic exhaustion serves as a precursor to stable functional exhaustion marked by increased expression of inhibitory receptors such as PD-1. The close relationship between metabolic rewiring and immune checkpoint regulation further reinforces this suppressive state, as metabolic alterations can regulate checkpoint expression in both tumor and immune compartments [135]. Thus, metabolic deprivation imposed by glioma cells—through nutrient sequestration, extracellular acidification, and production of inhibitory metabolites such as lactate—constitutes a central driver of effector T-cell dysfunction, energetic collapse, and broader immune suppression, representing a major barrier to successful immunotherapy [136,137].

### 7.2. Direct Immunomodulation by Neurotransmitter Signaling

Infiltrating T cells within brain tumors can be exposed to neurotransmitter-rich microenvironments that influence antitumor activity. As noted above, GABA receptor signaling may suppress CD8^+^ T-cell proliferation and cytotoxicity, but direct glioma-specific validation remains limited [87]. The glutamatergic axis is better supported in glioma models: pharmacological reduction in intratumoral glutamate with BHV-4157 decreases glutamate levels, increases CD4^+^ T-cell infiltration, reduces Foxp3+ regulatory T cells, and synergizes with anti-PD-1 therapy; this benefit is lost after the depletion of CD4^+^ or CD8^+^ T cells, confirming a T-cell-dependent component [138]. System-level modeling also identifies glutamate uptake inhibition in T cells as a potential strategy to counter glioma-associated metabolic suppression [139]. These findings indicate that neurotransmitter signaling can constrain effector immunity, although the degree of direct versus indirect action on T cells remains pathway-specific [140,141].

Consequently, effector T cells within gliomas are subjected to a dual inhibitory pressure: metabolic deprivation, which imposes nutrient starvation, and neurotransmitter-mediated suppression, which functionally restrains or paralyzes immune activity [87,138,139]. This convergence of starvation and signaling-dependent inhibition profoundly weakens the adaptive immune response and likely contributes to the characteristic resistance of gliomas to immunotherapeutic intervention. Collectively, these metabolic and neurotransmitter-dependent regulatory mechanisms converge to suppress effector immune responses within the glioma microenvironment, as summarized in Table 1.

## 8. Therapeutic Implications and Future Perspectives: Mechanism-Guided Targeting of Metabolic and Neurotransmitter Axes

### 8.1. Therapeutic Strategies Targeting Metabolic Reprogramming

Targeting the metabolic reprogramming intrinsic to gliomas represents a major therapeutic frontier aimed at dismantling the immunosuppressive tumor microenvironment (TME) and restoring antitumor immunity. A central strategy focuses on the inhibition of key enzymes and transporters involved in the Warburg phenotype, which is characterized by excessive glycolysis and sustained lactate production. Small-molecule inhibitors directed against lactate dehydrogenase A (LDHA) or monocarboxylate transporters (MCTs), including the MCT1 inhibitor AZD3965, are currently under clinical investigation [3]. The therapeutic rationale is to reverse the profound extracellular acidification caused by lactate efflux, which impairs cytotoxic T-cell function and promotes polarization of tumor-associated macrophages and microglia (TAMs) toward an immunosuppressive M2-like phenotype [3]. By limiting lactate export, these agents aim to alleviate microenvironmental acidosis and restore the metabolic fitness and effector capacity of infiltrating immune cells.

Additional glycolytic nodes also represent attractive targets. Inhibition of phosphofructo-2-kinase/fructose-2,6-bisphosphatase 3 (PFKFB3), for example, with PFK15, has shown promising activity in preclinical diffuse midline glioma models by inducing apoptosis and cell-cycle arrest [143]. Beyond glycolysis, therapeutic efforts also target amino acid metabolic pathways that simultaneously support tumor growth and deprive immune cells of essential substrates. The inhibition of IDO1 and TDO2 seeks to reduce tryptophan depletion and kynurenine accumulation, thereby attenuating the suppressive pathways restraining T-cell activity [66,144]. Similarly, targeting glutaminolysis with glutaminase inhibitors such as CB-839 aims to disrupt a key anaplerotic dependency in glutamine-reliant glioma subtypes and has already been explored in combinatorial settings [145,146].

Importantly, these metabolic interventions are unlikely to achieve maximal efficacy as standalone therapies and are more plausibly positioned as sensitizing components of combination regimens with immunotherapies such as immune checkpoint inhibitors (ICIs). The underlying logic is mechanistically coherent: metabolic modulators can reverse the “starved” and functionally “paralyzed” state of T cells within the TME, thereby creating a permissive metabolic environment in which reactivated lymphocytes can exert cytotoxic function in response to anti-PD-1/PD-L1-directed therapies [147,148]. This principle is exemplified by preclinical studies in IDH-mutant gliomas, where the inhibition of the oncometabolite D-2-hydroxyglutarate (D-2HG), when combined with radiotherapy, temozolomide, and anti-PD-L1 treatment, resulted in complete tumor regression in a substantial proportion of mice, accompanied by reduced T-cell exhaustion and enhanced generation of memory CD8^+^ T cells [147]. Taken together, these findings indicate that targeting metabolic reprogramming is evolving from a purely tumor-centric intervention into a multilayered immunorestorative strategy capable of reshaping the glioma microenvironment and improving the therapeutic impact of contemporary immunotherapy. Representative therapeutic strategies targeting metabolic pathways that contribute to immunosuppression within the glioma tumor microenvironment are summarized in Table 2.

### 8.2. Therapeutic Potential of Intervening in Neurotransmitter Signaling Co-Option

Co-option of neurotransmitter signaling by glioma cells represents a compelling therapeutic vulnerability. Intervening in these pathways offers an opportunity to disrupt tumor-promoting neural circuitry and, potentially, to remodel the immunosuppressive microenvironment. One major strategy involves antagonizing excitatory glutamatergic signaling. Excessive glutamate release, frequently facilitated by system Xc^−^ (xCT), contributes to tumor progression and glioma-associated epilepsy by establishing neuronal hyperexcitability [165,166]. NMDA receptor antagonists such as memantine or glutamate-release inhibitors such as sulfasalazine may therefore limit excitotoxic damage and neural activity-driven tumor support [167]. However, the clinical restoration of immune balance by memantine in glioma has not been demonstrated; any immune benefit should currently be regarded as a mechanistic hypothesis or repurposing rationale.

A parallel but less mature strategy involves modulation of inhibitory GABAergic signaling. GABA can suppress cytotoxic T-cell function and alter myeloid behavior in experimental systems [86,87], while broader cancer-stress literature supports neuroendocrine and neurotransmitter regulation of immune surveillance [106]. Nevertheless, glioma-specific therapeutic evidence for GABA receptor antagonists remains early. These agents should therefore be described as conceptual or preclinical candidates whose utility will depend on receptor subtype, sex, myeloid context, seizure status, and potential effects on normal inhibitory neurotransmission [168].

An upstream therapeutic approach focuses on transporters that mediate aberrant neurotransmitter accumulation and release. Because xCT was described mechanistically in Section 4.1, the therapeutic point is summarized here: inhibition of xCT by sulfasalazine or more selective next-generation compounds may reduce glutamate export, weaken tumor antioxidant capacity, and modulate the neural–tumor ecosystem [165,166,169]. Beyond xCT, transporters involved in tryptophan and tyrosine handling may indirectly reshape neurochemical availability and tumor–immune interactions [169]. Representative strategies targeting neurotransmitter-mediated immunoregulatory pathways are summarized in Table 3, with developmental status indicated to distinguish repurposing concepts from validated clinical effects.

### 8.3. Translational Strategies for Dual-Axis Targeting

The current standard of care for the treatment of GBM is defined by the Stupp protocol, which involves maximal safe surgical resection followed by concurrent radiotherapy and temozolomide (TMZ)-based chemotherapy and, subsequently, 6–12 months of adjuvant TMZ treatment [181]. Although this regimen significantly improved patient survival, therapeutic resistance and profound tumor-associated immunosuppression continue to represent major clinical challenges. Interestingly, the first major breakthrough since the introduction of the Stupp protocol in 2005 emerged through targeting metabolic rewiring with vorasidenib, a dual inhibitor of mutant IDH1 and IDH2 enzymes that suppresses production of the oncometabolite D-2-hydroxyglutarate (D-2HG). In the phase III INDIGO trial, progression-free survival increased from 11.1 months in the placebo arm to 27.7 months in the vorasidenib-treated group, corresponding to a 61% reduction in the risk of progression or death, emphasizing that targeting metabolic reprogramming may constitute a clinically effective therapeutic strategy capable of substantially delaying glioma progression and counteracting tumor-supportive mechanisms driven by metabolic dysregulation [182].

While these findings highlight the therapeutic potential of targeting metabolic rewiring in gliomas, abnormal neurotransmitter signaling and pathological neuron–glioma interactions may represent another therapeutically exploitable feature of GBM biology. Talampanel, an AMPA receptor antagonist, was evaluated with radiotherapy and temozolomide in newly diagnosed GBM and separately in recurrent high-grade glioma [183,184]. The newly diagnosed study reported encouraging survival compared with historical expectations and acceptable tolerability, but it was a single-arm phase II trial without a concurrent control group. In recurrent GBM, outcomes were more limited. Therefore, talampanel should be interpreted as providing early clinical feasibility for glutamatergic pathway modulation rather than definitive evidence of clinical benefit.

The GLUGLIO trial represents a clinically relevant attempt to test glutamate signaling inhibition in combination with standard chemoradiotherapy. It is a phase Ib/II randomized, open-label protocol comparing gabapentin, sulfasalazine, memantine, and chemoradiotherapy with chemoradiotherapy alone in newly diagnosed GBM [185,186]. Importantly, published information currently establishes trial design rather than efficacy. Accordingly, GLUGLIO should be cited as an example of ongoing clinical evaluation of glutamatergic pathway modulation in combination with standard therapy, rather than as evidence of clinical efficacy or validated dual-axis targeting.

#### 8.3.1. Biomarker-Guided Dual-Axis Intervention

The rationale for dual-axis intervention rests on three observations: gliomas exploit metabolic pathways such as tryptophan catabolism, adenosine signaling, and arginine depletion to establish immune tolerance; neuron-to-tumor synaptic communication and neurotransmitter signaling can drive proliferation and immune exclusion; and selected interactions between these axes may create microenvironments that exhaust infiltrating T cells and undermine immunotherapy [142,149,170,187]. Because the strength of evidence varies across mechanisms, biomarker-guided strategies should match specific patients to specific actionable nodes rather than broadly labeling all combinations as dual-axis therapies.

Translational success, therefore, requires not only the identification of actionable nodes within each axis but also the development of biomarker frameworks capable of matching patients to mechanism-informed therapeutic combinations and enabling immune effector cells to function within metabolically hostile tumor niches. Precision implementation of dual-axis strategies begins with molecular stratification aimed at identifying patients whose tumors exhibit specific metabolic vulnerabilities.

Tryptophan metabolism represents a tractable immunometabolic checkpoint. Tumor expression and activity of indoleamine 2,3-dioxygenase 1 (IDO1) and tryptophan 2,3-dioxygenase (TDO) drive immune tolerance and may serve as selection biomarkers for tryptophan-axis inhibitors or combinational immunometabolic approaches [150,151]. Patients with elevated IDO1/TDO expression may therefore represent candidates for interventions aimed at restoring tryptophan availability and relieving kynurenine-mediated T-cell suppression.

Arginine metabolism provides a complementary stratification axis. Loss of argininosuccinate synthase 1 (ASS1) expression defines a subset of gliomas characterized by arginine auxotrophy, potentially predicting sensitivity to arginine-directed therapeutic strategies [158]. However, arginine depletion carries important immunomodulatory tradeoffs, as arginine is essential for T-cell proliferation and effector function. This necessitates careful patient selection and potentially sequential or compartmentalized delivery strategies capable of sparing systemic immune function while targeting tumor arginine dependence [158].

Adenosine pathway activity, reflected by the expression of ectonucleotidases CD39 and CD73 or adenosine receptor-associated signatures, may further identify tumors that exploit adenosine-mediated immunosuppression [159]. Multi-parametric metabolic profiling integrating transcriptomics with metabolite imaging, including glucose, glutamine, and lipid utilization, may help prioritize metabolic inhibitors for tumors with dominant nutrient dependencies and guide the selection of mechanism-informed combination strategies [160,162,163].

Neurotransmitter-axis stratification relies on identifying tumor dependence on neuronal signaling. Elevated expression of glutamate receptor subunits, particularly AMPAR-associated components such as GRIA2 and GRIA3, may identify tumors dependent on neuron–tumor communication and potentially responsive to AMPAR antagonists or alternative neurotransmitter-modulating therapies. Glutamatergic signaling through AMPARs contributes to neuron–glioma synaptic activity and immune exclusion, positioning AMPAR blockade as a rational adjuvant to immunotherapy in biomarker-selected patients [171].

In parallel, pharmacologic neurotransmitter modulation, including repurposed agents such as selective serotonin reuptake inhibitors (SSRIs) and serotonin-norepinephrine reuptake inhibitors (SNRIs), has demonstrated synergy with targeted kinase inhibition in preclinical diffuse midline glioma models, illustrating a potential translational route for neurotransmitter-axis drug repurposing.

Importantly, multi-parametric stratification integrating both metabolic and neurotransmitter-related biomarkers may enable rational selection of mechanism-informed combinations while minimizing antagonistic effects. For example, arginine deprivation strategies require careful assessment of immune consequences, whereas AMPK activation combined with glutamate uptake inhibition has been identified through systems-level modeling as a potentially high-impact strategy for reversing tumor-mediated T-cell suppression [188]. The sex-specific GABBR-gMDSC pathway described above further suggests that sex, myeloid subtype, and arginine/NOS2 programs may become relevant stratification variables for future studies [105]. Adaptive trial designs incorporating biomarker-guided escalation or de-escalation of therapeutic components may help optimize therapeutic windows while minimizing toxicity.

#### 8.3.2. Metabolic Engineering of CAR-T Cells for Glioma Immunotherapy

The metabolically hostile glioma microenvironment, characterized by glucose depletion, lactate accumulation, amino acid imbalance, and neurotransmitter-associated stress, can impair CAR-T-cell trafficking, persistence, and effector function. For this review, the key point is not CAR-T engineering in solid tumors generally, but how CAR-T cells might be adapted to survive the specific metabolic–neurotransmitter constraints of glioma.

One cell-intrinsic strategy is to enhance metabolic fitness. Overexpression of glucose transporters GLUT1 and GLUT3 in CD70-targeted CAR-T cells improves glucose uptake, intratumoral trafficking, persistence, and antitumor activity under glucose-restricted conditions [173,174]. In glioma, such engineering could be paired with patient selection for highly glycolytic or lactate-rich tumors, where nutrient competition is expected to be most severe.

A second strategy is to protect CAR-T cells from suppressive signaling within neurometabolic niches. CRISPR-enabled engineering may combine improved oxidative metabolism, reduced exhaustion, resistance to checkpoint-mediated inhibition, and adaptation to amino acid scarcity [175,176]. Future designs could also test whether glutamate transport modulation, AMPK pathway activation, or resistance to GABA-associated suppression improves CAR-T function in glioma-specific models rather than generic solid tumor assays.

A third strategy is microenvironmental remodeling. Checkpoint-switch constructs such as PD1-CD28 fusion receptors convert inhibitory PD-L1 signaling into costimulatory activation [177,178], while CAR-T cells engineered to secrete SIRPγ-derived CD47 blockers may recruit phagocytic myeloid populations [179]. These approaches are most relevant to the dual-axis framework when combined with metabolic or neurotransmitter-directed interventions that make the glioma niche more permissive to immune effector function.

Thus, future CAR-T studies should explicitly test whether metabolic engineering, glutamate pathway modulation, or targeting sex- and myeloid-specific neurotransmitter programs improves immune persistence within glioma. This would align CAR-T development more closely with the neurometabolic framework proposed in this review. Accordingly, early-phase studies combining CAR-T therapy with carefully selected metabolic or neurotransmitter-axis modulators may benefit from biomarker-guided patient stratification based on indicators of glucose competition, lactate accumulation, AMPK activity, glutamate metabolism, and myeloid suppressive programs.

### 8.4. Prospects and Challenges of Multi-Target Combination Therapy

Given the extensive, but incompletely resolved, interaction between metabolic and neural signaling pathways within the glioma immune microenvironment, simultaneous targeting of selected nodes may offer therapeutic benefit. However, not every microenvironmental co-target should be considered part of the core metabolic reprogramming axis. For example, adenosine-A2AR signaling and CXCL12/CXCR4-mediated immune exclusion are best viewed as immunosuppressive niche-modifying pathways that can be combined with metabolic or neurotransmitter-directed interventions, rather than as direct components of lactate or nutrient competition biology [161,189]. Emerging and alternative combination strategies, including selected traditional Chinese medicine-derived compounds that may influence metabolic or neuroimmune pathways, further illustrate that multi-target approaches may arise from diverse therapeutic sources, although rigorous glioma-specific validation remains essential [190].

More broadly, specific forms of metabolic reprogramming—including alterations in tyrosine, tryptophan, and lactate metabolism—are linked to PD-L1 expression and immunosuppressive TME formation, providing justification for intervention at defined nodes [154,191,192]. Dysregulation of copper, fatty acid, and cholesterol metabolism in glioma-associated immune populations, particularly macrophages, contributes to T-cell exhaustion and immunotherapy resistance, identifying additional vulnerabilities suitable for combination strategies [155,156,193]. Integration of metabolic therapies with radiotherapy or ferroptosis induction represents another dimension of treatment. For example, paeonol has been reported to inhibit human glioma cell growth by inducing ferroptosis, providing a glioma-specific example of ferroptosis as an antitumor metabolic intervention, although its immune consequences remain to be defined [152,157,164,194,195,196].

The rationale for co-targeting neural pathways should also be framed precisely. Glutamatergic signaling, xCT-dependent glutamate export, and neuron–glioma synaptic activity support tumor expansion and glioma-associated epilepsy, while GABA-associated metabolic signaling may influence motility and immune regulation [165,172,197,198]. Proteomic studies further identify glioma subgroups such as a “metabolic–neural” class enriched for both metabolic enzymes and neurotransmitter receptor proteins, supporting biological coherence between these axes [180]. These findings justify mechanism-guided co-targeting, but they do not yet establish a universal dual-axis therapy for all glioma patients.

At the same time, the development of such strategies faces substantial challenges. A major obstacle remains the blood–brain barrier (BBB), which markedly limits penetration of many candidate agents into the central nervous system [154]. This makes advanced delivery systems essential. Approaches under investigation include biomimetic nanoplatforms camouflaged with hybrid glioma–macrophage membranes to improve BBB penetration and tumor selectivity [194], engineered outer membrane vesicles for coordinated payload delivery [195], and intracavitary sprayable hydrogel systems for localized postoperative treatment [156]. Another major concern is the potential effect of these interventions on normal brain physiology, especially cognition, given the essential functions of metabolites such as lactate and neurotransmitters such as glutamate and GABA in normal neuronal and synaptic activity [192,197]. For example, inhibition of xCT or glutamatergic signaling, while potentially antitumorigenic, must be balanced against the risk of worsening neuronal excitotoxicity or disrupting physiologic neurotransmission [72,165].

Tumor heterogeneity and adaptive compensation represent additional limitations. Gliomas display profound inter- and intratumoral heterogeneity at genetic, metabolic, and proteomic levels, resulting in variable therapeutic responses and rapid adaptive escape [1,180]. Targeting a single metabolic enzyme, such as mutant IDH, for example, may be circumvented through alternative metabolic pathways or epigenetic adaptation [153]. Likewise, the immunosuppressive TME is sustained by multiple redundant circuits; blockade of one pathway, such as adenosine signaling, may be insufficient if parallel suppressive systems, including tryptophan metabolism or myeloid-driven regulation, remain intact [199,200]. This complexity is reflected in the spatial distribution of glioma subtypes, which correlates with regional brain properties such as neurotransmitter receptor density and functional connectivity, suggesting that local microenvironmental context may shape therapeutic vulnerability [201].

Future research will need to integrate advanced models and technologies to overcome these constraints and fully exploit the potential of multi-target therapy. Patient-derived organoid co-cultures, humanized mouse models, and spatially resolved single-cell multi-omics will be essential for recapitulating the human glioma metabolism–neuroimmune network and for testing rational combinations [1,180,202]. These systems should specifically determine which interactions are direct, which are correlative, and which are context-dependent. Brain-targeted delivery systems capable of sequential or simultaneous release, responsiveness to TME cues, and precise extracellular or intracellular targeting will also be needed to minimize off-tumor effects [189,196,203,204].

Ultimately, precision implementation should stratify patients on the basis of integrated molecular profiles that include metabolic subtype, neural interaction signatures, immune contexture, and validated therapeutic vulnerabilities. Such stratification is a framework for patient selection, not itself a therapy. It may guide the rational selection of regimens tailored to vulnerabilities such as tryptophan metabolism, arginine dependence, lactate-rich niches, or AMPAR-associated neurotransmitter phenotypes [154,172,191,205].

## 9. Conclusions

In conclusion, glioma-associated immunosuppression is shaped by both metabolic reprogramming and neurotransmitter signaling co-option. The strongest evidence supports lactate-driven acidosis, nutrient competition, tryptophan/kynurenine signaling, and glutamatergic neuron–glioma communication as major contributors to immune dysfunction. Evidence for a fully bidirectional neurometabolic circuit is promising but still incomplete, particularly for GABAergic macrophage polarization and serotonergic immunometabolic mechanisms in glioma.

The value of the dual-axis framework is therefore conceptual and translational: it encourages studies that test how metabolic stress, neural activity, and immune dysfunction interact within spatially defined glioma niches. Future therapeutic development should move beyond broad combination language and instead pair specific biomarkers with specific interventions, such as tryptophan-axis inhibition, lactate/MCT targeting, glutamatergic pathway modulation, or metabolically optimized cellular therapy.

At the same time, clinical translation must remain cautious. Many proposed interventions are preclinical, repurposing-based, or supported by indirect evidence from non-glioma models. Effective implementation will require brain-penetrant agents, careful protection of normal neurotransmission, spatially informed patient selection, and trials designed to distinguish feasibility from genuine efficacy. A clearer mechanistic map of the metabolic–neural–immune interface may ultimately support safer and more effective strategies for reversing glioma-associated immune suppression.

## Figures and Tables

**Table 1:** Metabolic and neurotransmitter-associated mechanisms of immunosuppression in the glioma tumor microenvironment: mechanisms, functional effects, and evidence context.

Axis	Tumor-Associated Factor/Process	Target Immune Cells	Mechanism	Functional Outcome	Evidence/Model Context	References
Lactate metabolism	Lactate	CD8^+^ T cells	Inhibition of mTORC1 signaling	Reduced IFN-γ production and cytotoxicity	Glioma-specific evidence and experimental T-cell models	[123,130]
Glycolytic shift	Acidic TME	Effector T cells	Suppression of TCR signaling	Functional exhaustion	Experimental acidic TME and immune cell models, with supporting glioma-related evidence	[123,126,130]
Methionine metabolism	Methionine depletion	T cells	Epigenetic restriction of activation programs	Reduced proliferation and activation	Experimental T-cell metabolic models and glioma-associated metabolic evidence	[128]
Arginine metabolism	Arginine depletion	T cells	Impaired metabolic fitness	Reduced expansion and effector function	Glioma-related studies and experimental immune cell models	[69,129,142]
Lactate signaling	Lactate accumulation	Tregs	Promotion of a regulatory phenotype	Increased immunosuppression	Experimental Treg and tumor microenvironment models, including acidic TME studies	[51,52]
Glutamate signaling	Extracellular glutamate	CD4^+^ and CD8^+^ T cells	Disruption of metabolic homeostasis	Reduced immune cell infiltration and antitumor responses	Glioma datasets and experimental glioma-associated immune models	[137]
GABA signaling	GABA	CD8^+^ T cells	Receptor-mediated immune suppression	Reduced proliferation and effector activity	Primarily non-glioma tumor and experimental immune cell models; direct glioma validation remains limited	[99,100,101,105]
Dopamine signaling	DRD2 activation	Macrophages	PD-L1 upregulation and polarization shift	Enhanced immunosuppressive phenotype	Experimental macrophage and tumor models; glioma-specific evidence remains context-dependent	[120,141]

**Table 2:** Therapeutic strategies and biomarker-guided translational frameworks targeting metabolic and immunometabolic drivers of immunosuppression in the glioma tumor microenvironment.

Translational Category	Pathway Axis	Molecular Target/Process	Example Therapeutic Strategy	Expected Immune/Microenvironmental Effect	Level of Translational Development	References
**Clinical and clinically relevant strategies**	Lactate transport	MCT1	AZD3965	Inhibition of lactate transport and attenuation of lactate-dependent metabolic immune suppression	Clinical investigation	[47]
	Tryptophan metabolism	IDO1/TDO2	IDO1 and/or TDO2 inhibition	Reduction in kynurenine-mediated T-cell suppression and restoration of effector immune responses	Clinical evaluation, including combination strategies	[65,66,149,150,151]
**Preclinical and experimental strategies**	Lactate production	LDHA	LDHA inhibition	Reduction in lactate accumulation and extracellular acidosis, with restoration of cytotoxic T-cell activity and attenuation of suppressive GAM polarization	Preclinical and early translational	[3,59,61]
	Glycolytic regulation	PFKFB3	PFKFB3 inhibition with PFK15	Induction of tumor cell apoptosis and cell-cycle arrest, with potential indirect improvement of immune permissiveness	Preclinical	[143]
	Glutamine metabolism	Glutaminase	CB-839	Disruption of tumor anaplerosis and potential improvement of immune cell metabolic fitness	Preclinical and combination-based evaluation	[145,146]
	Oncometabolite signaling	Mutant IDH-dependent D-2HG production	Inhibition of mutant IDH/D-2HG signaling combined with radiotherapy, temozolomide, or anti-PD-L1 therapy	Reduction in T-cell exhaustion and promotion of memory CD8^+^ T-cell responses	Preclinical combination therapy	[147,152,153]
	Metabolic regulation of immune checkpoints	Lactate-, kynurenine-, and amino acid-dependent regulation of PD-L1	Combination of metabolic pathway inhibition with immune checkpoint blockade	Reduction in immune checkpoint expression and improved responsiveness to immunotherapy	Preclinical and early translational evidence	[71,135,154]
	Lipid and cholesterol immunometabolism	Fatty acid and cholesterol metabolism in tumor-associated macrophages	Inhibition or reprogramming of macrophage lipid metabolism	Reduction in macrophage-mediated immune suppression and T-cell dysfunction	Emerging preclinical strategy	[13,155,156]
	Ferroptosis-associated metabolism	Ferroptosis regulatory pathways	Paeonol-induced ferroptosis	Inhibition of glioma growth through ferroptotic cell death; immune consequences remain to be established	Preclinical	[157]
	Adenosine-associated immunometabolic signaling	CD73–A2AR axis	Inhibition of CD73–A2AR signaling	Reversal of adenosine-mediated immune suppression and restoration of antitumor immune activity	Preclinical adjunctive strategy	[158,159,160,161]
**Biomarker-guided translational frameworks**	Metabolic stratification	IDO1/TDO2- and ASS1-associated signatures	Biomarker-guided patient selection and trial design	Identification of patients most likely to benefit from metabolism-directed combination therapies	Conceptual translational framework; not a therapeutic intervention	[150,151,162,163,164]

**Table 3:** Therapeutic strategies and biomarker-guided translational frameworks targeting neurotransmitter-associated and dual-axis mechanisms in the glioma tumor microenvironment.

Translational Category	Pathway Axis	Molecular Target/Process	Example Therapeutic Strategy	Expected Immune/Microenvironmental Effect	Level of Translational Development	References
**Clinical and clinically relevant strategies**	Glutamatergic signaling	NMDA receptor activity	Memantine	Reduction in excitotoxic glutamatergic signaling and neuronal activity-driven tumor support; immune benefit in glioma remains unproven	Clinically approved drug; repurposing rationale	[84,85]
**Preclinical and experimental strategies**	Glutamate release and redox regulation	System Xc^−^/xCT transporter	Sulfasalazine or more selective next-generation xCT inhibitors	Reduction in glutamate export, neuronal hyperexcitability, and tumor antioxidant capacity, with potential modulation of the neural–tumor microenvironment	Preclinical and drug-repurposing rationale; next-generation inhibitors remain experimental	[73,85,165,169]
	AMPAR-mediated neuron–glioma signaling	AMPA receptor signaling	AMPAR antagonism or inhibition of downstream signaling	Disruption of neuron–glioma synaptic communication and attenuation of activity-dependent tumor support; immune consequences require further validation	Experimental and emerging	[77,78,170,171]
	GABAergic immunoregulation	GABA receptor signaling	GABA receptor antagonists or subtype-selective modulators	Potential attenuation of GABA-associated suppression of T-cell and myeloid-cell activity; direct therapeutic evidence in glioma remains limited	Conceptual and early-stage; requires glioma-specific validation	[99,100,101,105,168]
	Amino acid transporter modulation	Tryptophan and tyrosine transport pathways	Transporter-directed modulation	Alteration of neurotransmitter precursor availability and potential reshaping of tumor–immune interactions; glioma-specific therapeutic validation remains limited	Emerging experimental strategy	[75,169]
	Metabotropic glutamate signaling	mGlu receptor signaling	Subtype-selective mGlu receptor modulation	Potential attenuation of tumor-supportive glutamatergic signaling and modulation of immune infiltration; evidence remains experimental and receptor-subtype-dependent	Experimental	[140,172]
**Preclinical and early translational strategies**	Dual-axis immunotherapy optimization	GLUT1/GLUT3 expression, AMPK activity, glutamate handling, and checkpoint-switch receptors	Metabolically engineered CAR-T cells combined with resistance to suppressive microenvironmental signals	Improved CAR-T metabolic fitness, persistence, and effector activity within the nutrient-depleted and neurochemically active glioma microenvironment	Preclinical and early translational	[173,174,175,176,177,178,179]
**Biomarker-guided translational frameworks**	Integrated metabolic–neurotransmitter stratification	IDO1/TDO2-, ASS1-, and GRIA2/GRIA3-associated signatures	Biomarker-guided patient selection and trial design	Identification of molecularly defined patient subgroups most likely to benefit from mechanism-based metabolic and neurotransmitter-directed combinations	Translational framework; not a therapeutic intervention	[150,151,162,163,164,180]
	Sex-specific neuroimmune stratification	GABBR–gMDSC/NOS2-associated programs	Biomarker-guided selection of sex- and myeloid-context-dependent therapeutic strategies	Identification of biologically distinct immunosuppressive subgroups and potential responders to neurotransmitter-directed interventions	Emerging translational framework; not a therapeutic intervention	[105]

## Data Availability

No new data were created or analyzed in this study. Data sharing is not applicable to this article.

## References

[1] Sharma P., Aaroe A., Liang J., Puduvalli V.K. (2023). Tumor microenvironment in glioblastoma: Current and emerging concepts. Neuro-Oncol. Adv..

[2] Malhotra D., Gabrani R. (2025). Metabolic shifts in glioblastoma: Unraveling altered pathways and exploring novel therapeutic avenues. Mol. Biol. Rep..

[3] Xie X., Zhou W., Ku Y., Li S., Yang Y., Hao X., Chen Y. (2025). Lactate-Mediated Epigenetic and Immunometabolic Reprogramming in Glioma: An Emerging Axis Linking Metabolism to Tumor Progression. Biomedicines.

[4] Wang S., Huang T., Wu Q., Yuan H., Wu X., Yuan F., Duan T., Taori S., Zhao Y., Snyder N.W. (2024). Lactate reprograms glioblastoma immunity through CBX3-regulated histone lactylation. J. Clin. Investig..

[5] Ugolini A., De Leo A., Yu X., Scirocchi F., Liu X., Peixoto B., Scocozza D., Pace A., Perego M., Gardini A. (2025). Functional Reprogramming of Neutrophils within the Brain Tumor Microenvironment by Hypoxia-Driven Histone Lactylation. Cancer Discov..

[6] He C., Sheng L., Pan D., Jiang S., Ding L., Ma X., Liu Y., Jia D. (2021). Single-Cell Transcriptomic Analysis Revealed a Critical Role of SPP1/CD44-Mediated Crosstalk Between Macrophages and Cancer Cells in Glioma. Front. Cell Dev. Biol..

[7] Yuile A., Wei J.Q., Mohan A.A., Hotchkiss K.M., Khasraw M. (2023). Interdependencies of the Neuronal, Immune and Tumor Microenvironment in Gliomas. Cancers.

[8] Sun R., Kim A.H. (2022). The multifaceted mechanisms of malignant glioblastoma progression and clinical implications. Cancer Metastasis Rev..

[9] Wen J., Wu X., Shu Z., Wu D., Yin Z., Chen M., Luo K., Liu K., Shen Y., Le Y. (2025). Clusterin-mediated polarization of M2 macrophages: A mechanism of temozolomide resistance in glioblastoma stem cells. Stem Cell Res. Ther..

[10] Liu H., Sun Y., Zhang Q., Jin W., Gordon R.E., Zhang Y., Wang J., Sun C., Wang Z.J., Qi X. (2021). Pro-inflammatory and proliferative microglia drive progression of glioblastoma. Cell Rep..

[11] Rao R., Han R., Ogurek S., Xue C., Wu L.M., Zhang L., Zhang L., Hu J., Phoenix T.N., Waggoner S.N. (2022). Glioblastoma genetic drivers dictate the function of tumor-associated macrophages/microglia and responses to CSF1R inhibition. Neuro-Oncology.

[12] Pombo Antunes A.R., Scheyltjens I., Duerinck J., Neyns B., Movahedi K., Van Ginderachter J.A. (2020). Understanding the glioblastoma immune microenvironment as basis for the development of new immunotherapeutic strategies. eLife.

[13] Governa V., de Oliveira K.G., Bång-Rudenstam A., Offer S., Cerezo-Magaña M., Li J., Beyer S., Johansson M.C., Månsson A.-S., Edvardsson C. (2024). Protumoral lipid droplet-loaded macrophages are enriched in human glioblastoma and can be therapeutically targeted. Sci. Transl. Med..

[14] Liu X., Song Z., Sun Z., Liu C., Kang X., Qiao H., Tu X., Li T., Fu Z., Wang Y. (2026). *MSTN* and *TCF12* as Candidate Immunometabolic Signatures in Glioma-Associated Foam Cells: Insights from Integrated Multi-Omics Analysis. Curr. Issues Mol. Biol..

[15] Ghouzlani A., Kandoussi S., Tall M., Reddy K.P., Rafii S., Badou A. (2021). Immune Checkpoint Inhibitors in Human Glioma Microenvironment. Front. Immunol..

[16] Rosato P.C., Wijeyesinghe S., Stolley J.M., Nelson C.E., Davis R.L., Manlove L.S., Pennell C.A., Blazar B.R., Chen C.C., Geller M.A. (2019). Virus-specific memory T cells populate tumors and can be repurposed for tumor immunotherapy. Nat. Commun..

[17] Sarantopoulos A., Ene C., Aquilanti E. (2024). Therapeutic approaches to modulate the immune microenvironment in gliomas. npj Precis. Oncol..

[18] Zhao J., Wu D., Liu J., Zhang Y., Li C., Zhao W., Cao P., Wu S., Li M., Li W. (2024). Disease-specific suppressive granulocytes participate in glioma progression. Cell Rep..

[19] Clavreul A., Menei P. (2020). Mesenchymal Stromal-Like Cells in the Glioma Microenvironment: What Are These Cells?. Cancers.

[20] Cai X., Yuan F., Zhu J., Yang J., Tang C., Cong Z., Ma C. (2021). Glioma-Associated Stromal Cells Stimulate Glioma Malignancy by Regulating the Tumor Immune Microenvironment. Front. Oncol..

[21] Goldberg A.R., Dovas A., Torres D., Pereira B., Viswanathan A., Das Sharma S., Mela A., Merricks E.M., Megino-Luque C., McInvale J.J. (2024). Glioma-induced alterations in excitatory neurons are reversed by mTOR inhibition. bioRxiv.

[22] Faisal S.M., Comba A., Varela M.L., Argento A.E., Brumley E., Abel C., Castro M.G., Lowenstein P.R. (2022). The complex interactions between the cellular and non-cellular components of the brain tumor microenvironmental landscape and their therapeutic implications. Front. Oncol..

[23] Ulasov I., Singh V., Laevskaya A., Timashev P., Kharwar R.K. (2023). Inflammatory Mediators and GBM Malignancy: Current Scenario and Future Prospective. Discov. Med..

[24] Elguindy M.M., Young J.S., Ho W.S., O Lu R.O. (2024). Co-evolution of glioma and immune microenvironment. J. Immunother. Cancer.

[25] Hansen L.J., Jackson C.M. (2025). The glioma microenvironment and its impact on antitumor immunity. Neuro-Oncol. Adv..

[26] Robilliard L.D., Yu J., Anchan A., Joseph W., Finlay G., E Angel C., Graham E.S. (2021). Comprehensive analysis of inhibitory checkpoint ligand expression by glioblastoma cells. Immunol. Cell Biol..

[27] Lin H., Liu C., Hu A., Zhang D., Yang H., Mao Y. (2024). Understanding the immunosuppressive microenvironment of glioma: Mechanistic insights and clinical perspectives. J. Hematol. Oncol..

[28] Ma T., Su G., Wu Q., Shen M., Feng X., Zhang Z. (2024). Tumor-derived extracellular vesicles: How they mediate glioma immunosuppression. Mol. Biol. Rep..

[29] Musatova O., Kumar V., Vinogradov K., Rubtsov Y. (2025). Immune checkpoints in immune response to glioma: Two sides of the same coin. Front. Immunol..

[30] Krishna S., Choudhury A., Keough M.B., Seo K., Ni L., Kakaizada S., Lee A., Aabedi A., Popova G., Lipkin B. (2023). Glioblastoma remodelling of human neural circuits decreases survival. Nature.

[31] Nejo T., Krishna S., Yamamichi A., Lakshmanachetty S., Jimenez C., Lee K.Y., Baker D.L., Young J.S., Chen T., Phyu S.S.S. (2025). Glioma-neuronal circuit remodeling induces regional immunosuppression. Nat. Commun..

[32] Akl C.F., Andersen B.M., Li Z., Giovannoni F., Diebold M., Sanmarco L.M., Kilian M., Fehrenbacher L., Pernin F., Rone J.M. (2025). Glioblastoma-instructed astrocytes suppress tumour-specific T cell immunity. Nature.

[33] Andersen B.M., Faust Akl C.F., Wheeler M.A., Li Z., Diebold M., Kilian M., Rone J.M., Misra A., Kenison J.E., Lee J.-H. (2025). Barcoded viral tracing identifies immunosuppressive astrocyte-glioma interactions. Nature.

[34] Li F., Han Y., Ou F., Deng L., Li H., Yu X., Yi Y., Ma R., Wu Z., You Z. (2026). Spatial heterogeneity of MDSCs mediated by ANXA1-FPRs signaling drives immune suppression in OSCC progression. Nat. Commun..

[35] Ludwig N., Rao A., Sandlesh P., Yerneni S.S., Swain A.D., Bullock K.M., Hansen K.M., Zhang X., Jaman E., Allen J. (2022). Characterization of systemic immunosuppression by IDH mutant glioma small extracellular vesicles. Neuro-Oncology.

[36] Wang M., Cai Y., Peng Y., Xu B., Hui W., Jiang Y. (2020). Exosomal LGALS9 in the cerebrospinal fluid of glioblastoma patients suppressed dendritic cell antigen presentation and cytotoxic T-cell immunity. Cell Death Dis..

[37] Friedrich M., Sankowski R., Bunse L., Kilian M., Green E., Guevara C.R., Pusch S., Poschet G., Sanghvi K., Hahn M. (2021). Tryptophan metabolism drives dynamic immunosuppressive myeloid states in IDH-mutant gliomas. Nat. Cancer.

[38] Wang L., Zhang H., Jing X., Huang Y., Kang Y., Qian Y., Chen D. (2025). The oncometabolite R-2-hydroxyglutarate inhibits microglial activation via the FTO/NF-κB pathway. Front. Oncol..

[39] Wang Z., Dai Z., Zhang H., Liang X., Zhang X., Wen Z., Luo P., Zhang J., Liu Z., Zhang M. (2023). Tumor-secreted lactate contributes to an immunosuppressive microenvironment and affects CD8 T-cell infiltration in glioblastoma. Front. Immunol..

[40] Liu X., Zhou Y., Wang H. (2024). The role of lactate-induced protein lactylation in gliomas: Implications for preclinical research and the development of new treatments. Front. Pharmacol..

[41] Schurr A. (2025). Glioma neuron symbiosis: A hypothesis. Front. Neurosci..

[42] Samad A., Samant R., Rao K.V., Bhargava V., I Sadique S., Sadique S.I., Sr. K.V.R., Yadav R.Y. (2023). Oxaloacetate as a Holy Grail Adjunctive Treatment in Gliomas: A Revisit to Metabolic Pathway. Cureus.

[43] Paech D., Nagel A.M., Schultheiss M.N., Umathum R., Regnery S., Scherer M., Wick A., Platt T., Wick W., Bendszus M. (2020). Quantitative Dynamic Oxygen 17 MRI at 7.0 T for the Cerebral Oxygen Metabolism in Glioma. Radiology.

[44] Autry A.W., Vaziri S., LaFontaine M., Gordon J.W., Chen H.-Y., Kim Y., Villanueva-Meyer J.E., Molinaro A., Clarke J.L., Bush N.A.O. (2023). Multi-parametric hyperpolarized 13C/1H imaging reveals Warburg-related metabolic dysfunction and associated regional heterogeneity in high-grade human gliomas. NeuroImage Clin..

[45] Pagano C., Coppola L., Navarra G., Avilia G., Savarese B., Torelli G., Bruzzaniti S., Piemonte E., Galgani M., Laezza C. (2024). N6-isopentenyladenosine inhibits aerobic glycolysis in glioblastoma cells by targeting PKM2 expression and activity. FEBS Open Bio.

[46] Guda M.R., Tsung A.J., Asuthkar S., Velpula K.K. (2022). Galectin-1 activates carbonic anhydrase IX and modulates glioma metabolism. Cell Death Dis..

[47] Miranda-Gonçalves V., Gonçalves C.S., Granja S., de Castro J.V., Reis R.M., Costa B.M., Baltazar F. (2021). MCT1 Is a New Prognostic Biomarker and Its Therapeutic Inhibition Boosts Response to Temozolomide in Human Glioblastoma. Cancers.

[48] Jiang M., Wang Y., Zhao X., Yu J. (2024). From metabolic byproduct to immune modulator: The role of lactate in tumor immune escape. Front. Immunol..

[49] Zuo L., Zhao Y., Huang Z., Wang D., Xu F., Zhang M., He S., Yang K. (2026). A Biomimetic Microparticle Disrupting the Intracellular/Extracellular pH Homeostasis of Tumor Cells for Cancer Chemo-Immunotherapy. ACS Nano.

[50] Rahman M.A., Yadab M.K., Ali M.M. (2024). Emerging Role of Extracellular pH in Tumor Microenvironment as a Therapeutic Target for Cancer Immunotherapy. Cells.

[51] Tuomela K., Levings M.K. (2023). Acidity promotes the differentiation of immunosuppressive regulatory T cells. Eur. J. Immunol..

[52] Mani N.L., Weinberg S.E., Chaudhuri S., Montauti E., Tang A., Iyer R., Fang D. (2024). Acidity induces durable enhancement of Treg cell suppressive functions for tumor immune evasion. Mol. Immunol..

[53] Sunil H.S., Clemenceau J.R., Grichuk A., Barnfather I., Nakkireddy S.R., Izzo L., Feng Q., Hartnett W., Evers B.M., Thomas L. (2025). TMPRSS11B promotes an acidified microenvironment and immune suppression in squamous lung cancer. EMBO Rep..

[54] Jakobsen E., Bech J.M., Andersen J.V., Westi E.W., Larsen M.R., Skotte N.H., Moreira J.M., Aldana B.I., Bak L.K. (2025). Deletion of AC8 in glioma cells elevates oxidative phosphorylation by system-wide remodeling of the mitochondrial proteome. Biochim. Biophys. Acta Bioenerg..

[55] Tian L.-R., Lin M.-Z., Zhong H.-H., Cai Y.-J., Li B., Xiao Z.-C., Shuai X.-T. (2022). Nanodrug regulates lactic acid metabolism to reprogram the immunosuppressive tumor microenvironment for enhanced cancer immunotherapy. Biomater. Sci..

[56] Knudsen-Clark A.M., Mwangi D., Cazarin J., Morris K., Baker C., Hablitz L.M., McCall M.N., Kim M., Altman B.J. (2024). Circadian rhythms of macrophages are altered by the acidic pH of the tumor microenvironment. bioRxiv.

[57] Liu R., Ren X., Park Y.E., Feng H., Sheng X., Song X., AminiTabrizi R., Shah H., Li L., Zhang Y. (2025). Nuclear GTPSCS functions as a lactyl-CoA synthetase to promote histone lactylation and gliomagenesis. Cell Metab..

[58] Zhao J., Liu X., He Y., Sun Q., Xue Z., Tang Z., Liu J., Wang J., Li C., Wang X. (2026). Histone H4K8 lactylation promotes glioblastoma progression by inducing NUPR1-mediated autophagosome-lysosome fusion. Theranostics.

[59] Barba I., Carrillo-Bosch L., Seoane J. (2024). Targeting the Warburg Effect in Cancer: Where Do We Stand?. Int. J. Mol. Sci..

[60] Sun Y., Wang H., Cui Z., Yu T., Song Y., Gao H., Tang R., Wang X., Li B., Li W. (2025). Lactylation in cancer progression and drug resistance. Drug Resist. Updat..

[61] Liang X.H., Chen X.Y., Yan Y., Cheng A., Lin J., Jiang Y., Chen H., Jin J., Luan X. (2024). Targeting Metabolism to Enhance Immunotherapy within Tumor Microenvironment. Acta Pharmacol. Sin..

[62] Coleman M.F., Cozzo A.J., Pfeil A.J., Etigunta S.K., Hursting S.D. (2020). Cell Intrinsic and Systemic Metabolism in Tumor Immunity and Immunotherapy. Cancers.

[63] Qiu Y., Xu Y., Ding X., Zhao C., Cheng H., Li G. (2025). Bi-directional metabolic reprogramming between cancer cells and T cells reshapes the anti-tumor immune response. PLoS Biol..

[64] Niu Y., Mayr T., Muders M.H. (2021). Competition for nutrients or cell intrinsic programming?—Metabolic mechanisms behind the tumor promoting immune microenvironment in cancer. Signal Transduct. Target. Ther..

[65] Platten M., Friedrich M., A Wainwright D., Panitz V., A Opitz C. (2021). Tryptophan metabolism in brain tumors — IDO and beyond. Curr. Opin. Immunol..

[66] Kudo T., Prentzell M.T., Mohapatra S.R., Sahm F., Zhao Z., Grummt I., Wick W., Opitz C.A., Platten M., Green E.W. (2020). Constitutive Expression of the Immunosuppressive Tryptophan Dioxygenase TDO2 in Glioblastoma Is Driven by the Transcription Factor C/EBPβ. Front. Immunol..

[67] Hu S., Heng H., Yang F., Wang M., Liu G., Xiang Y., Miao H. (2025). The metabolism-immune axis in colorectal cancer: Remodeling the tumor microenvironment through metabolite signaling. Front. Immunol..

[68] Corrado M., Frezza C. (2023). Glutamine availability unleashes dendritic cells’ anti-tumor power. Cell Chem. Biol..

[69] Pilanc P., Wojnicki K., Roura A.-J., Cyranowski S., Ellert-Miklaszewska A., Ochocka N., Gielniewski B., Grzybowski M.M., Błaszczyk R., Stańczak P.S. (2021). A Novel Oral Arginase 1/2 Inhibitor Enhances the Antitumor Effect of PD-1 Inhibition in Murine Experimental Gliomas by Altering the Immunosuppressive Environment. Front. Oncol..

[70] Wei F., Wang D., Wei J., Tang N., Tang L., Xiong F., Guo C., Zhou M., Li X., Li G. (2021). Metabolic crosstalk in the tumor microenvironment regulates antitumor immunosuppression and immunotherapy resisitance. Cell. Mol. Life Sci..

[71] Yin Z., Bai L., Li W., Zeng T., Tian H., Cui J. (2019). Targeting T cell metabolism in the tumor microenvironment: An anti-cancer therapeutic strategy. J. Exp. Clin. Cancer Res..

[72] Yakubov E., Schmid S., Hammer A., Chen D., Dahlmanns J.K., Mitrovic I., Zurabashvili L., Savaskan N., Steiner H.-H., Dahlmanns M. (2023). Ferroptosis and PPAR-gamma in the limelight of brain tumors and edema. Front. Oncol..

[73] Park J.W., Kilic O., Deo M., Jimenez-Cowell K., Demirdizen E., Kim H., Turcan Ş. (2023). CIC reduces xCT/SLC7A11 expression and glutamate release in glioma. Acta Neuropathol. Commun..

[74] Feyissa A.M., Rosenfeld S.S., Quiñones-Hinojosa A. (2022). Altered glutamatergic and inflammatory pathways promote glioblastoma growth, invasion, and seizures: An overview. J. Neurol. Sci..

[75] Umans R.A., Martin J., Harrigan M.E., Patel D.C., Chaunsali L., Roshandel A., Iyer K., Powell M.D., Oestreich K., Sontheimer H. (2021). Transcriptional Regulation of Amino Acid Transport in Glioblastoma Multiforme. Cancers.

[76] Cueto-Ureña C., Ramírez-Expósito M.J., Martínez-Martos J.M. (2025). Neuron-Glioma Synapses in Tumor Progression. Biomedicines.

[77] Radin D.P. (2025). AMPA Receptor Modulation in the Treatment of High-Grade Glioma: Translating Good Science into Better Outcomes. Pharmaceuticals.

[78] Taylor K.R., Barron T., Hui A., Spitzer A., Yalçin B., Ivec A.E., Geraghty A.C., Hartmann G.G., Arzt M., Gillespie S.M. (2023). Glioma synapses recruit mechanisms of adaptive plasticity. Nature.

[79] Anastasaki C., Mu R., Kernan C.M., Li X., Barakat R., Koleske J.P., Gao Y., Cobb O.M., Lu X., Eberhart C.G. (2025). Aberrant coupling of glutamate and tyrosine kinase receptors enables neuronal control of brain-tumor growth. Neuron.

[80] Wirsching H.G., Weller M. (2020). Does Neuronal Activity Promote Glioma Progression?. Trends Cancer.

[81] Lange F., Hörnschemeyer M.F., Kirschstein T. (2021). Glutamatergic Mechanisms in Glioblastoma and Tumor-Associated Epilepsy. Cells.

[82] Grimi A., Bono B.C., Lazzarin S.M., Marcheselli S., Pessina F., Riva M. (2024). Gliomagenesis, Epileptogenesis, and Remodeling of Neural Circuits: Relevance for Novel Treatment Strategies in Low- and High-Grade Gliomas. Int. J. Mol. Sci..

[83] Hatcher A., Yu K., Meyer J., Aiba I., Deneen B., Noebels J.L. (2020). Pathogenesis of peritumoral hyperexcitability in an immunocompetent CRISPR-based glioblastoma model. J. Clin. Investig..

[84] Lee R.X. (2026). Inhibitory glutamatergic feedback for brain tumor therapy. Med. Oncol..

[85] Kumaria A., Ashkan K. (2023). Novel therapeutic strategies in glioma targeting glutamatergic neurotransmission. Brain Res..

[86] Mondal J., Huse J.T. (2025). Neurotransmitter power plays: The synaptic communication nexus shaping brain cancer. Acta Neuropathol. Commun..

[87] Xu L., Chen S., Fu Y., Zhou T., Yu J., Li J., Chen W. (2025). Neuro-immune-tumor axis in gliomas: A review of mechanisms, models, and translational opportunities. Front. Immunol..

[88] Nguyen H.D., Diamandis P., Scott M.S., Richer M. (2022). Deciphering of Adult Glioma Vulnerabilities through Expression Pattern Analysis of GABA, Glutamate and Calcium Neurotransmitter Genes. J. Pers. Med..

[89] Cen W., Fu G., Wang X., Wei R., Zhou X., Teng W., Ling Y., Tang J., Wang Z., Chu L. (2025). GABRP Mediates GABA-A Receptor to Shape Tumor Immunosuppressive Microenvironment and Promote Tumor Immune Escape and Corresponding Targeted Therapy. Cancer Med..

[90] Weidner L., Lorenz J., Quach S., Braun F.K., Rothhammer-Hampl T., Ammer L.-M., Vollmann-Zwerenz A., Bartos L.M., Dekorsy F.J., Holzgreve A. (2023). Translocator protein (18kDA) (TSPO) marks mesenchymal glioblastoma cell populations characterized by elevated numbers of tumor-associated macrophages. Acta Neuropathol. Commun..

[91] Ismail F.S., Faustmann P.M., Corvace F., Faustmann T.J. (2025). Neuroglia in autoimmune encephalitis. Handb. Clin. Neurol..

[92] Prener M., Opheim G., Shams Z., Søndergaard C.B., Lindberg U., Larsson H.B.W., Ziebell M., Larsen V.A., Vestergaard M.B., Paulson O.B. (2023). Single-Voxel MR Spectroscopy of Gliomas with s-LASER at 7T. Diagnostics.

[93] Jiang Y., Lan Y., Wang Y., Chen S., Shen Y., Chu S., Dong Y., Li L., Zhang H., Lu Z. (2026). Integrative Multi-Omics Analysis Reveals the Characteristic Metabolic Signature of Glioma and Enables Plasma-Based Liquid Biopsy. Research.

[94] Bale A.A., Thammineni S., Bhargava R., Harley B. (2024). Hyaluronic Acid Influences Amino Acid Metabolism via Differential L-Type Amino Acid Transporter 1 Expression in the U87-Malignant Glioma Cell Line. Adv. NanoBiomed Res..

[95] Rich L.J., Bagga P., Wilson N.E., Schnall M.D., Detre J.A., Haris M., Reddy R. (2020). 1H magnetic resonance spectroscopy of 2H-to-1H exchange quantifies the dynamics of cellular metabolism in vivo. Nat. Biomed. Eng..

[96] Skredėnienė R., Stakišaitis D., Valančiūtė A., Balnytė I. (2025). In Vivo and In Vitro Experimental Study Comparing the Effect of a Combination of Sodium Dichloroacetate and Valproic Acid with That of Temozolomide on Adult Glioblastoma. Int. J. Mol. Sci..

[97] Fu J., Han Z., Wu Z., Xia Y., Yang G., Yin Y., Ren W. (2022). GABA regulates IL-1β production in macrophages. Cell Rep..

[98] Xia Y., He F., Wu X., Tan B., Chen S., Liao Y., Qi M., Chen S., Peng Y., Yin Y. (2021). GABA transporter sustains IL-1β production in macrophages. Sci. Adv..

[99] Huang D., Wang Y., Thompson J.W., Yin T., Alexander P.B., Qin D., Mudgal P., Wu H., Liang Y., Tan L. (2022). Cancer-cell-derived GABA promotes β-catenin-mediated tumour growth and immunosuppression. Nat. Cell Biol..

[100] Tang D., Orlandi P., Li Q., Bandini A., Bocci G. (2025). GABAergic signaling in colorectal cancer: Mechanistic insights, tumor microenvironment crosstalk, and therapeutic opportunities. Biochim. Biophys. Acta Rev. Cancer.

[101] Zhou X., Chen Z., Yu Y., Li M., Cao Y., Prochownik E.V., Li Y. (2024). Increases in 4-Acetaminobutyric Acid Generated by Phosphomevalonate Kinase Suppress CD8^+^ T Cell Activation and Allow Tumor Immune Escape. Adv. Sci..

[102] Zhao J., Yang Z., Tu M., Meng W., Gao H., Li M.D., Li L. (2021). Correlation Between Prognostic Biomarker SLC1A5 and Immune Infiltrates in Various Types of Cancers Including Hepatocellular Carcinoma. Front. Oncol..

[103] Wang X., Fu S.-Q., Yuan X., Yu F., Ji Q., Tang H.-W., Li R.-K., Huang S., Huang P.-Q., Qin W.-T. (2024). A GAPDH serotonylation system couples CD8+ T cell glycolytic metabolism to antitumor immunity. Mol. Cell.

[104] Zhang Y., Wang N., Zhang L., Zhuang Y., Xin Q., Gu X., Jiang C., Wu J. (2025). Serotonin (5-Hydroxytryptamine): Metabolism, Signaling, Biological Functions, Diseases, and Emerging Therapeutic Opportunities. Medcomm.

[105] Pathak A., Palasalava S., Knott M.V., Colon B., Ciervo E., Zhou Y., Mitchell J., Teran Pumar O., Wong H.K.A., Zhang L. (2024). γ-aminobutyric acid receptor B signaling drives glioblastoma in females in an immune-dependent manner. bioRxiv.

[106] Huang B., An H., Wu H., Qiu Y., Su Y., Chen L., Georgakopoulou V.E., Lin J., Chen W., Li R. (2025). Chronic Psychological Stress in Oncogenesis: Multisystem Crosstalk and Multimodal Interventions. Research.

[107] Li Z.K., Liao J.L., Luo M.R., Fang S.J., Huang W.Z., Zhang D.Y. (2025). Neuro-tumor interactions: Multi-dimensional mechanisms of neurotransmitter regulation in tumor immune evasion and metabolic reprogramming. World J. Clin. Oncol..

[108] Miyajima M. (2020). Amino acids: Key sources for immunometabolites and immunotransmitters. Int. Immunol..

[109] Sowers M.L., Tang H., Singh V.K., Khan A., Mishra A., Restrepo B.I., Jagannath C., Zhang K. (2022). Multi-OMICs analysis reveals metabolic and epigenetic changes associated with macrophage polarization. J. Biol. Chem..

[110] Vijayanathan Y., Ho I.A.W. (2025). The Impact of Metabolic Rewiring in Glioblastoma: The Immune Landscape and Therapeutic Strategies. Int. J. Mol. Sci..

[111] Zhao X., Ren T., Li S., Wang X., Hou R., Guan Z., Liu D., Zheng J., Shi M. (2024). A new perspective on the therapeutic potential of tumor metastasis: Targeting the metabolic interactions between TAMs and tumor cells. Int. J. Biol. Sci..

[112] Liu X., Yu Q. (2025). Advances in tumor-associated macrophage-mediated chemotherapeutic resistance in glioma. Front. Cell Dev. Biol..

[113] Du Y., Yang Y., Zhang C., Yuan Q. (2026). The spatiotemporal dynamic evolution of post-stroke neuroinflammation: Energy metabolism mechanisms of acute response and chronic progression. Front. Pharmacol..

[114] Wu L., Zhang X., Zheng L., Zhao H., Yan G., Zhang Q., Zhou Y., Lei J., Zhang J., Wang J. (2020). RIPK3 Orchestrates Fatty Acid Metabolism in Tumor-Associated Macrophages and Hepatocarcinogenesis. Cancer Immunol. Res..

[115] Lu Y., Sun Q., Guan Q., Zhang Z., He Q., He J., Ji Z., Tian W., Xu X., Liu Y. (2023). The XOR-IDH3α axis controls macrophage polarization in hepatocellular carcinoma. J. Hepatol..

[116] Jin X., Zhang N., Yan T., Wei J., Hao L., Sun C., Zhao H., Jiang S. (2025). Lactate-mediated metabolic reprogramming of tumor-associated macrophages: Implications for tumor progression and therapeutic potential. Front. Immunol..

[117] Li S., Lu E., Mi L., Li C., Guan H., Cao T., Zhang Q. (2025). Research on the interactive mechanisms between mitochondrial variations and immune responses in gliomas based on integrated visualization analysis. Discov. Oncol..

[118] Dong Y., Wang G., Nie D., Xu Y., Bai X., Lu C., Jian F., Wang H., Zheng X. (2023). Tumor-derived GABA promotes lung cancer progression by influencing TAMs polarization and neovascularization. Int. Immunopharmacol..

[119] Blyufer A., Lhamo S., Tam C., Tariq I., Thavornwatanayong T., Mahajan S.S. (2021). Riluzole: A neuroprotective drug with potential as a novel anti-cancer agent (Review). Int. J. Oncol..

[120] Liu L., Wu Y., Wang B., Jiang Y., Lin L., Li X., Yang S. (2021). DA-DRD5 signaling controls colitis by regulating colonic M1/M2 macrophage polarization. Cell Death Dis..

[121] Gan Z., Zhang M., Xie D., Wu X., Hong C., Fu J., Fan L., Wang S., Han S. (2021). Glycinergic Signaling in Macrophages and Its Application in Macrophage-Associated Diseases. Front. Immunol..

[122] Yuan D., Hu J., Ju X., Putz E.M., Zheng S., Koda S., Sun G., Deng X., Xu Z., Nie W. (2023). NMDAR antagonists suppress tumor progression by regulating tumor-associated macrophages. Proc. Natl. Acad. Sci. USA.

[123] Reuss A.M., Groos D., Buchfelder M., Savaskan N. (2021). The Acidic Brain-Glycolytic Switch in the Microenvironment of Malignant Glioma. Int. J. Mol. Sci..

[124] Watson M.J., Delgoffe G.M. (2022). Fighting in a wasteland: Deleterious metabolites and antitumor immunity. J. Clin. Investig..

[125] Minami N., Tanaka K., Sasayama T., Kohmura E., Saya H., Sampetrean O. (2021). Lactate Reprograms Energy and Lipid Metabolism in Glucose-Deprived Oxidative Glioma Stem Cells. Metabolites.

[126] Lim A.R., Rathmell W.K., Rathmell J.C. (2020). The tumor microenvironment as a metabolic barrier to effector T cells and immunotherapy. eLife.

[127] Zhou Z., Xu J., Huang N., Tang J., Ma P., Cheng Y. (2022). Clinical and Biological Significance of a Necroptosis-Related Gene Signature in Glioma. Front. Oncol..

[128] Hu H., Huang Y., Li J., Liu R., Li H., Cai M., Chen H., Wang N., Yang S., Wang K. (2025). Glioma promotes macrophage immunosuppressive phenotype through ANXA1 in a methionine metabolism-dependent manner. Discov. Oncol..

[129] Hou X., Chen S., Zhang P., Guo D., Wang B. (2022). Targeted Arginine Metabolism Therapy: A Dilemma in Glioma Treatment. Front. Oncol..

[130] Qiu R., Zhong Y., Li Q., Li Y., Fan H. (2021). Metabolic Remodeling in Glioma Immune Microenvironment: Intercellular Interactions Distinct From Peripheral Tumors. Front. Cell Dev. Biol..

[131] Trejo-Solís C., Castillo-Rodríguez R.A., Serrano-García N., Silva-Adaya D., Vargas-Cruz S., Chávez-Cortéz E.G., Gallardo-Pérez J.C., Zavala-Vega S., Cruz-Salgado A., Magaña-Maldonado R. (2024). Metabolic Roles of HIF1, c-Myc, and p53 in Glioma Cells. Metabolites.

[132] Zhou J., Li L., Jia M., Liao Q., Peng G., Luo G., Zhou Y. (2023). Dendritic cell vaccines improve the glioma microenvironment: Influence, challenges, and future directions. Cancer Med..

[133] Sun T., Li Y., Wu J., Cao Y., Yang Y., He Y., Huang W., Liu B., Yang W. (2022). Downregulation of exosomal MHC-I promotes glioma cells escaping from systemic immunosurveillance. Nanomedicine.

[134] Koss B., Shields B.D., Taylor E.M., Storey A.J., Byrum S.D., Gies A.J., Washam C.L., Choudhury S.R., Ahn J.H., Uryu H. (2020). Epigenetic Control of *Cdkn2a.Arf* Protects Tumor-Infiltrating Lymphocytes from Metabolic Exhaustion. Cancer Res..

[135] Kumar A., Chamoto K. (2021). Immune metabolism in PD-1 blockade-based cancer immunotherapy. Int. Immunol..

[136] Grabowski M.M., Sankey E.W., Ryan K.J., Chongsathidkiet P., Lorrey S.J., Wilkinson D.S., Fecci P.E. (2021). Immune suppression in gliomas. J. Neuro-Oncol..

[137] Fan H., Yang S., Lu Q., Chang L. (2026). Metabolic reprogramming and immunosenescence: A new sight for glioma therapy. Front. Cell Dev. Biol..

[138] Medikonda R., Choi J., Pant A., Saleh L., Routkevitch D., Tong L., Belcaid Z., Kim Y.H., Jackson C.M., Jackson C. (2022). Synergy between glutamate modulation and anti-programmed cell death protein 1 immunotherapy for glioblastoma. J. Neurosurg..

[139] Shukla M., Bhowmick R., Ganguli P., Sarkar R.R. (2024). Metabolic reprogramming and signalling cross-talks in tumour-immune interaction: A system-level exploration. R. Soc. Open Sci..

[140] Fan H., Yan D., Fang X., Xiao L., Liang M., Wu H., Zhu G., Geng D., Liu Q. (2024). Low expression of GRM4 is associated with poor prognosis and tumor immune infiltration in glioma. Int. J. Neurosci..

[141] Liu Y.-S., Huang B.-R., Lin C.-J., Shen C.-K., Lai S.-W., Chen C.-W., Lin H.-J., Lin C.-H., Hsieh Y.-C., Lu D.-Y. (2021). Paliperidone Inhibits Glioblastoma Growth in Mouse Brain Tumor Model and Reduces PD-L1 Expression. Cancers.

[142] Yuxiao C., Jiachen W., Yanjie L., Shenglan L., Yuji W., Wenbin L. (2024). Therapeutic potential of arginine deprivation therapy for gliomas: A systematic review of the existing literature. Front. Pharmacol..

[143] de Camargo Magalhães E.S.d.C., de Bont E.S., Bruggeman S.W.M., Lima F.R.S. (2024). Targeting the reprogrammed metabolism in H3.3K27M pediatric high-grade gliomas. Biochim. Biophys. Acta Mol. Basis Dis..

[144] Mohiuddin M.K., Thakar S., Pradhan S.S., Rajaratnam S., Kanikaram S.P., Pulukool S.K., Sivaramakrishnan V., Mohan D. (2026). Serum Metabolomic Signature of Gliomas in an Indian Cohort: Identification of Grade-Specific Alterations and Potential Biomarkers. Neurol. India.

[145] Fu H., Wu S., Shen H., Luo K., Huang Z., Lu N., Li Y., Lan Q., Xian Y. (2024). Glutamine Metabolism Heterogeneity in Glioblastoma Unveils an Innovative Combination Therapy Strategy. J. Mol. Neurosci..

[146] Guo Y., Jiang T., Liang S., Wang A., Li J., Jia Y., Li Q., Yin J., Bai S., Li J. (2025). Immunostimulatory Hydrogel with Synergistic Blockage of Glutamine Metabolism and Chemodynamic Therapy for Postoperative Management of Glioblastoma. Adv. Sci..

[147] Kadiyala P., Carney S.V., Gauss J.C., Garcia-Fabiani M.B., Haase S., Alghamri M.S., Núñez F.J., Liu Y., Yu M., Taher A. (2021). Inhibition of 2-hydroxyglutarate elicits metabolic reprogramming and mutant IDH1 glioma immunity in mice. J. Clin. Investig..

[148] Jabri A., Mhannayeh A., Taftafa B., Alsharif M., Sibai D., Alsharif R., Abbad T., Elsalti A., Ahmed Z., Salma J. (2025). Recent advances in immunotherapy for gliomas: Overcoming barriers and advancing precision strategies. Front. Immunol..

[149] Du L., Xing Z., Tao B., Li T., Yang D., Li W., Zheng Y., Kuang C., Yang Q. (2020). Both IDO1 and TDO contribute to the malignancy of gliomas via the Kyn-AhR-AQP4 signaling pathway. Signal Transduct. Target. Ther..

[150] Wang X., Chen Z., Chen L., Qiu C. (2026). IDO family: The metabolic crossroads connecting immunity, neurological regulation and tumor microenvironment modulation. J. Transl. Med..

[151] Fiore A., Murray P.J. (2021). Tryptophan and indole metabolism in immune regulation. Curr. Opin. Immunol..

[152] Yan D., Li W., Liu Q., Yang K. (2022). Advances in Immune Microenvironment and Immunotherapy of Isocitrate Dehydrogenase Mutated Glioma. Front. Immunol..

[153] Han S., Liu Y., Cai S.J., Qian M., Ding J., Larion M., Gilbert M.R., Yang C. (2020). IDH mutation in glioma: Molecular mechanisms and potential therapeutic targets. Br. J. Cancer.

[154] Wang J.Y., Dai X.T., Gao Q., Chang H., Zhang S., Shan C., He T. (2023). Tyrosine metabolic reprogramming coordinated with the tricarboxylic acid cycle to drive glioma immune evasion by regulating PD-L1 expression. Ibrain.

[155] Jiang F., Luo F., Zeng N., Mao Y., Tang X., Wang J., Hu Y., Wu C. (2022). Characterization of Fatty Acid Metabolism-Related Genes Landscape for Predicting Prognosis and Aiding Immunotherapy in Glioma Patients. Front. Immunol..

[156] Dong Y., Zhang J., Wang Y., Zhang Y., Rappaport D., Yang Z., Han M., Liu Y., Fu Z., Zhao X. (2024). Intracavitary Spraying of Nanoregulator-Encased Hydrogel Modulates Cholesterol Metabolism of Glioma-Supportive Macrophage for Postoperative Glioblastoma Immunotherapy. Adv. Mater..

[157] Li X., Liao X.-Q., Chen W., Wu T., Li J., Wang L., He B., Qin X., Luo J. (2026). Paeonol Inhibits Cell Growth by Inducing Ferroptosis in Human Glioma Cells. Pharmacol. Res. Perspect..

[158] Xing J., Zhang J., Wang J. (2023). The Immune Regulatory Role of Adenosine in the Tumor Microenvironment. Int. J. Mol. Sci..

[159] Yan A., Joachims M.L., Thompson L.F., Miller A.D., Canoll P.D., Bynoe M.S. (2019). CD73 Promotes Glioblastoma Pathogenesis and Enhances Its Chemoresistance via A2B adenosine Receptor Signaling. J. Neurosci..

[160] Han Y., Dong C., Hu M., Wang X., Wang G. (2024). Unlocking the adenosine receptor mechanism of the tumor microenvironment. Front. Immunol..

[161] Gelsleichter N.E., Azambuja J.H., Rubenich D.S., Braganhol E. (2023). CD73 in glioblastoma: Where are we now and what are the future directions?. Immunol. Lett..

[162] Gokani V., Kotta A., Repaka V., Rauniyar S., Noch E.K. (2026). Targeting Metabolic Vulnerabilities in Glioblastoma: A Framework for Multi-node Combination Therapy. Curr. Oncol. Rep..

[163] Choudhary N., Osorio R.C., Oh J.Y., Aghi M.K. (2023). Metabolic Barriers to Glioblastoma Immunotherapy. Cancers.

[164] Yang G., Shan D., Zhao R., Li G. (2022). Metabolism-Associated DNA Methylation Signature Stratifies Lower-Grade Glioma Patients and Predicts Response to Immunotherapy. Front. Cell Dev. Biol..

[165] Divé I., A Schäfer J., Weber K.J., Çakır A.Y., A Verheyden N., I Lorenz N., Chong S.-A., Zeiner P.S., Franiczek C., Sauer B. (2026). Tumor-associated epilepsy and high expression of xCT shape the proteome of IDH-wildtype glioblastoma. Cell Death Discov..

[166] Șerban M., Toader C., Covache-Busuioc R.A. (2025). Brain Tumors, AI and Psychiatry: Predicting Tumor-Associated Psychiatric Syndromes with Machine Learning and Biomarkers. Int. J. Mol. Sci..

[167] Maier J.P., Ravi V.M., Kueckelhaus J., Behringer S.P., Garrelfs N., Will P., Sun N., von Ehr J., Goeldner J.M., Pfeifer D. (2021). Inhibition of metabotropic glutamate receptor III facilitates sensitization to alkylating chemotherapeutics in glioblastoma. Cell Death Dis..

[168] Ruffolo G., Alfano V., Romagnolo A., Zimmer T., Mills J.D., Cifelli P., Gaeta A., Morano A., Anink J., Mühlebner A. (2022). GABAA receptor function is enhanced by Interleukin-10 in human epileptogenic gangliogliomas and its effect is counteracted by Interleukin-1β. Sci. Rep..

[169] Srivastava S., Anbiaee R., Houshyari M., Sridhar S., Ashique S., Hussain S., Kumar S., Taj T., Akbarnejad Z., Taghizadeh-Hesary F. (2025). Amino acid metabolism in glioblastoma pathogenesis, immune evasion, and treatment resistance. Cancer Cell Int..

[170] Venkataramani V., Tanev D.I., Strahle C., Studier-Fischer A., Fankhauser L., Kessler T., Körber C., Kardorff M., Ratliff M., Xie R. (2019). Glutamatergic synaptic input to glioma cells drives brain tumour progression. Nature.

[171] Venkatesh H.S., Morishita W., Geraghty A.C., Silverbush D., Gillespie S.M., Arzt M., Tam L.T., Espenel C., Ponnuswami A., Ni L. (2019). Electrical and synaptic integration of glioma into neural circuits. Nature.

[172] Caridi M., Alborghetti M., Pellicelli V., Orlando R., Pontieri F.E., Battaglia G., Arcella A. (2024). Metabotropic Glutamate Receptors Type 3 and 5 Feature the “NeuroTransmitter-type” of Glioblastoma: A Bioinformatic Approach. Curr. Neuropharmacol..

[173] Sengupta S., Yamaguchi A., West I., DeYoung C., Rugger N., Jin D., Tao H., Chauhan A., Thomas N., Feier D. (2025). EXTH-109. Overexpressing glucose transporters to enhance CAR-T cell metabolic programming to treat glioblastoma. Neuro-Oncology.

[174] Shi Y., Kotchetkov I.S., Dobrin A., Hanina S.A., Rajasekhar V.K., Healey J.H., Sadelain M. (2024). GLUT1 overexpression enhances CAR T cell metabolic fitness and anti-tumor efficacy. Mol. Ther..

[175] McPhedran S.J., Carleton G.A., Lum J.J. (2024). Metabolic engineering for optimized CAR-T cell therapy. Nat. Metab..

[176] Wellhausen N., Agarwal S., Rommel P.C., I Gill S.I., June C.H. (2022). Better living through chemistry: CRISPR/Cas engineered T cells for cancer immunotherapy. Curr. Opin. Immunol..

[177] Hui E., Cheung J., Zhu J., Su X., Taylor M.J., Wallweber H.A., Sasmal D.K., Huang J., Kim J.M., Mellman I. (2017). T cell costimulatory receptor CD28 is a primary target for PD-1-mediated inhibition. Science.

[178] Chen W., Xian N., Zhao N., Zhang Q., Xu Y. (2024). PD1CD28 chimeric molecule enhances EGFRvIII-specific CAR-T cell activity against glioblastoma. PLoS ONE.

[179] Martins T.A., Kaymak D., Tatari N., Gerster F., Hogan S., Ritz M.-F., Sabatino V., Wieboldt R., Bartoszek E.M., McDaid M. (2024). Enhancing anti-EGFRvIII CAR T cell therapy against glioblastoma with a paracrine SIRPγ-derived CD47 blocker. Nat. Commun..

[180] Zhang J., Sun R., Lyu Y., Liu C., Liu Y., Feng Y., Fu M., Wong P.J.C., Du Z., Qiu T. (2024). Proteomic profiling of gliomas unveils immune and metabolism-driven subtypes with implications for anti-nucleotide metabolism therapy. Nat. Commun..

[181] Stupp R., Mason W.P., van den Bent M.J., Weller M., Fisher B., Taphoorn M.J.B., Belanger K., Brandes A.A., Marosi C., Bogdahn U. (2005). Radiotherapy plus Concomitant and Adjuvant Temozolomide for Glioblastoma. N. Engl. J. Med..

[182] Mellinghoff I.K., van den Bent M.J., Blumenthal D.T., Touat M., Peters K.B., Clarke J., Mendez J., Yust-Katz S., Welsh L., Mason W.P. (2023). Vorasidenib in IDH1- or IDH2-Mutant Low-Grade Glioma. N. Engl. J. Med..

[183] Grossman S.A., Ye X., Chamberlain M., Mikkelsen T., Batchelor T., Desideri S., Piantadosi S., Fisher J., Fine H.A. (2009). Talampanel With Standard Radiation and Temozolomide in Patients With Newly Diagnosed Glioblastoma: A Multicenter Phase II Trial. J. Clin. Oncol..

[184] Iwamoto F.M., Kreisl T.N., Kim L., Duic J.P., Butman J.A., Albert P.S., Fine H.A. (2010). Phase 2 trial of talampanel, a glutamate receptor inhibitor, for adults with recurrent malignant gliomas. Cancer.

[185] Wirsching H.G., Roth P., Fischer A., Hottinger A., Hundsberger T., Migliorini D., Ochsenbein A., Laeubli H., Seystahl K., Imbach L. (2023). RTID-04. Gluglio—A Phase IB/II Randomized Drug Repurposing Trial of Glutamate Signaling Inhibitors in Combination with Chemoradiotherapy in Patients with Newly Diagnosed Glioblastoma. Neuro-Oncology.

[186] Mastall M., Roth P., Bink A., Maranta A.F., Läubli H., Hottinger A.F., Hundsberger T., Migliorini D., Ochsenbein A., Seystahl K. (2024). A phase Ib/II randomized, open-label drug repurposing trial of glutamate signaling inhibitors in combination with chemoradiotherapy in patients with newly diagnosed glioblastoma: The GLUGLIO trial protocol. BMC Cancer.

[187] Keerthana C.K., Rayginia T.P., Shifana S.C., Anto N.P., Kalimuthu K., Isakov N., Anto R.J. (2023). The role of AMPK in cancer metabolism and its impact on the immunomodulation of the tumor microenvironment. Front. Immunol..

[188] Hong M., Baek J.H. (2025). Targeting AMPK for Cancer Therapy: Metabolic Reprogramming as a Therapeutic Strategy. Oncol. Res..

[189] Wei R., Xie K., Li T., Lin W., Zhao Y., Li J., Lai S., Wei X., Jiang X., Yuan Y. (2025). Immunity/metabolism dual-regulation via an acidity-triggered bioorthogonal assembly nanoplatform enhances glioblastoma immunotherapy by targeting CXCL12/CXCR4 and adenosine-A2AR pathways. Biomaterials.

[190] Lin Y., Chen Q., Dou X., Tang Y., Jin W., Zhang Y., Yu L. (2026). Research Progress in Immunotherapy and the Impact of Traditional Chinese Medicine on Glioblastoma. Curr. Neurovascular Res..

[191] Liu Y., Xiang J., Liao Y., Peng G., Shen C. (2022). Identification of tryptophan metabolic gene-related subtypes, development of prognostic models, and characterization of tumor microenvironment infiltration in gliomas. Front. Mol. Neurosci..

[192] Luo T., Liu L., Wang H., Wen S. (2025). Protein lactylation and immunotherapy in gliomas: A novel regulatory axis in tumor metabolism (Review). Int. J. Oncol..

[193] Luo Q., Zhuang J., Zheng D., Miao C., Luo H., Peng J., Zheng C., Qin C., Lan C., Chen M. (2023). IGFBP2 from a novel copper metabolism-associated biomarker promoted glioma progression and response to immunotherapy. Front. Immunol..

[194] Bai S., Li W., Yin N., Cao Y., Zhang S., Wang Z., Qu D., Wang B., Li Y., Liu X. (2026). Bi-Target Nanomodulator for Glioma-Associated Myeloid Cells Polarization by Inducing Pyroptosis and Regulating Tryptophan Metabolism to Reshape Immunosuppressive Microenvironment of IDH-Mutant Gliomas. Adv. Mater..

[195] Liang M., Fan L., Zhang S., Chen H., Dong X., Hu L., Wang G., Yang K., Pei P., Zhu R. (2025). 131I/MTX-loaded engineered outer membrane vesicles for sequentially coordinated radio-metabolic-immunotherapy of glioma. J. Nanobiotechnology.

[196] Wang B., Yin N., Liu X., Guan Y., Xu H., Yu Y., Bai Y., Cao Y., Wang Z., Bai S. (2026). Biomimetic sequentially gated nanotuners based on amorphous metal-organic frameworks for reprogramming the metabolism-ferroptosis-immunity crosstalk in gliomas. Mater. Today Bio.

[197] Wang Y., Wang Y., Bao H., Wang Z., Jiang T. (2025). Mechanisms and therapeutic implications of glioma-neuron interactions. Cancer Lett..

[198] Piperi C., Saurty-Seerunghen M.S., Levidou G., Sepsa A., Trigka E.-A., Klonou A., Markouli M., Strepkos D., Spyropoulou A., Kanakoglou D.S. (2023). Glioma Cells Expressing High Levels of ALDH5A1 Exhibit Enhanced Migration Transcriptional Signature in Patient Tumors. Neurotherapeutics.

[199] Wu J.Y., Ren A.L., Lim M. (2022). Immunometabolism, a new therapeutic development for immunotherapies of high-grade gliomas: A narrative review. Chin. Clin. Oncol..

[200] Lin Y.-J., Wu C.Y.-J., Wu J.Y., Lim M. (2022). The Role of Myeloid Cells in GBM Immunosuppression. Front. Immunol..

[201] Ren P., Bao H., Wang S., Wang Y., Bai Y., Lai J., Yi L., Liu Q., Li W., Zhang X. (2024). Multi-scale brain attributes contribute to the distribution of diffuse glioma subtypes. Int. J. Cancer.

[202] Wang S., Gao S., Lin S., Fang X., Zhang H., Qiu M., Zheng K., Ji Y., Xiao B., Zhang X. (2024). Integrated analysis of bulk and single-cell RNA sequencing reveals the impact of nicotinamide and tryptophan metabolism on glioma prognosis and immunotherapy sensitivity. BMC Neurol..

[203] Chen P., Zhao H., Gao X., Xu J., Huang Z., Shen H. (2025). Multi-Omics Analysis Unveils Nsun5-Mediated Molecular Alterations in the Somatosensory Cortex and Its Impact on Pain Sensation. Mol. Cell. Proteom..

[204] Dubiński P., Odzimek-Rajska M., Podlewski S., Brola W. (2025). Emerging Insights into the Role of the Microbiome in Brain Gliomas: A Systematic Review of Recent Evidence. Int. J. Mol. Sci..

[205] Chen S., Zhang S., Feng W., Li J., Yuan Y., Li W., Wang Z., Yang Y., Liu Y. (2022). Serine and glycine metabolism-related gene expression signature stratifies immune profiles of brain gliomas, and predicts prognosis and responses to immunotherapy. Front. Pharmacol..

